# Mechanistic insights from targeted molecular profiling of repolarization alternans in the intact human heart

**DOI:** 10.1093/europace/euz007

**Published:** 2019-02-08

**Authors:** Michele Orini, Joseph Yanni, Peter Taggart, Ben Hanson, Martin Hayward, Andrew Smith, Henggui Zhang, Michael Colman, Gareth Jones, Xiao Jie, Halina Dobrzynski, Mark R Boyett, Pier D Lambiase

**Affiliations:** 1 Institute of Cardiovascular Science, University College London, London, UK; 2 Department of Electrophysiology, Barts Heart Centre at St Bartholomew’s Hospital, London, UK; 3 Division of Cardiovascular Science, University of Manchester, Manchester, UK; 4 Department of Mechanical Engineering, University College London, UK; 5 Department of Cardiothoracic Surgery, The Heart Hospital, University College London Hospitals, London, UK; 6 School of Physics and Astronomy, University of Manchester, Manchester, UK; 7 School of Biomedical Sciences, University of Leeds, UK

**Keywords:** Arrhythmia mechanisms, Repolarization alternans, Sudden cardiac death, Whole heart cardiac mapping, Molecular profiling, Multi-electrode sock

## Abstract

**Aims:**

Action potential duration (APD) alternans is an established precursor or arrhythmia and sudden cardiac death. Important differences in fundamental electrophysiological properties relevant to arrhythmia exist between experimental models and the diseased *in vivo* human heart. To investigate mechanisms of APD alternans using a novel approach combining intact heart and cellular cardiac electrophysiology in human *in vivo*.

**Methods and results:**

We developed a novel approach combining intact heart electrophysiological mapping during cardiac surgery with rapid on-site data analysis to guide myocardial biopsies for laboratory analysis, thereby linking repolarization dynamics observed at the organ level with underlying ion channel expression. Alternans-susceptible and alternans-resistant regions were identified by an incremental pacing protocol. Biopsies from these sites (*n* = 13) demonstrated greater RNA expression in Calsequestrin (CSQN) and Ryanodine (RyR) and ion channels underlying *I*_K1_ and *I*_to_ at alternans-susceptible sites. Electrical restitution properties (*n* = 7) showed no difference between alternans-susceptible and resistant sites, whereas spatial gradients of repolarization were greater in alternans-susceptible than in alternans-resistant sites (*P* = 0.001). The degree of histological fibrosis between alternans-susceptible and resistant sites was equivalent. Mathematical modelling of these changes indicated that both CSQN and RyR up-regulation are key determinants of APD alternans.

**Conclusion:**

Combined intact heart and cellular electrophysiology show that regions of myocardium in the *in vivo* human heart exhibiting APD alternans are associated with greater expression of CSQN and RyR and show no difference in restitution properties compared to non-alternans regions. *In silico* modelling identifies up-regulation and interaction of CSQN with RyR as a major mechanism underlying APD alternans.

**What’s new?**

- We utilized a novel approach combining intact heart electrophysiological mapping, targeted myocardial biopsies for ion channel profiling, and computational modelling to study repolarization alternans, an established pro-arrhythmic marker, in the intact human heart.
- We identified up-regulation of two calcium cycling proteins (Calsequestrin and Ryanodine) as playing a major role in repolarization alternans development.
- This provides valuable human data to complement experimental studies in animal models, myocardial cells, and computational modelling.

## Introduction

Action potential duration (APD) alternans, an alternation of the APD on an every other beat basis, has long been recognized and linked to arrhythmogenesis.^1^ In the surface electrocardiogram (ECG) it manifests as microvolt T-wave alternans, a powerful non-invasive predictor,^2^^,^^3^ and precursor of potentially lethal ventricular arrhythmia.

The mechanism underlying repolarization alternans has been the subject of much investigation, as has the mechanism by which alternans may initiate arrhythmia. An important challenge remains to advance our understanding of the physiological and pathophysiological function of the human heart *in vivo* in order to combat the continuing high mortality due to cardiac arrhythmias. Whereas a great deal of vital information has been obtained from a range of experimental models, extrapolation from these models to the human is by no means straightforward owing to the well-known species differences in cardiomyocyte electrophysiology and species-dependent mechanisms underlying arrhythmias.^4^ Here, we have implemented a unique *in vivo* approach that enables the investigation of electrophysiological properties relevant to fatal arrhythmias. Myocardial biopsies, guided by rapid on-site analysis of intact heart electrophysiological recordings, enabled us to examine cellular properties in relation to intact heart behaviour. Detailed biophysical cellular and tissue models were then used to elucidate the mechanisms that link the identified cellular properties and electrophysiological abnormalities.

Repolarization alternans was initiated by a pacing induced increase in heart rate. Electrophysiological mapping identified areas of myocardium exhibiting APD alternans interspersed with areas showing no alternans. The cellular properties were compared from myocardial biopsies and restitution properties compared using a standard pacing protocol. The role of the cellular changes observed in the alternans regions in the generation of alternans was examined using biophysical modelling. Within this novel framework, our data provide evidence for the mechanisms underlying APD alternans in the intact human heart, and implicate calcium cycling mechanisms, specifically Calsequestrin (CSQN) interactions with Ryanodine (RyR), in the mechanism underlying APD alternans.

## Methods

A detailed description of the methods is provided in the Supplementary material online.

### Experimental protocol

Thirty-one subjects (age 62.5 ± 15.0 years, 28 male) were studied undergoing routine cardiac surgery for ischaemic heart disease (*n* = 22), aortic valve disease (*n* = 5), or both (*n* = 4). Most patients (71%) presented ischaemic cardiomyopathy with multiple vessels disease. Ten patients had a previous myocardial infarction, at least more than 12 months before enrolment. No patient had recent syncope or major arrhythmic event. Detailed patient information is shown in Supplementary material online, *Table S1*. The study was approved by the local ethics committee, and all subjects gave written informed consent.

**Figure 1:**
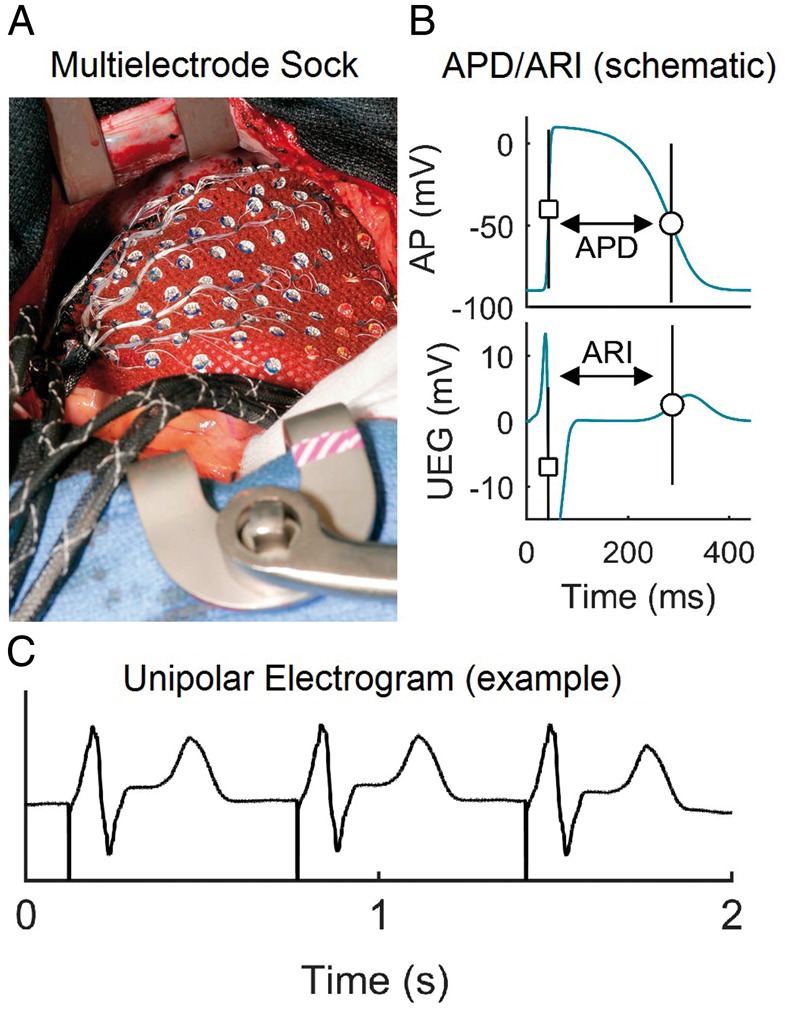
Experimental set-up. (*A*) Multi-electrode sock during open-heart surgery enabling the recording of 240 UEG. (*B*) Diagrammatic illustration of the relationship between APD and local ARI from UEG. (*C*) A representative unfiltered UEG. APD, action potential duration; ARI, activation-recovery interval; UEG, unipolar electrograms.

Following cannulation for cardiopulmonary bypass, a multi-electrode sock (240 electrodes) was fitted over the epicardium of both ventricles as described previously^5^ (*Figure 1*). Alternans was induced by incremental pacing. S_1_ drive trains of 30–50 beats were delivered from one of the electrodes of the multi-electrode sock at the right ventricular (RV) apex at cycle lengths decreasing from 600 ms to 350 ms. Unipolar electrograms were recorded at a sampling rate of 1 KHz and referenced to the rib retractor. Haemodynamic stability during the pacing protocol was closely monitored and pacing discontinued if appropriate. The electrograms were processed on-site and activation-recovery intervals (ARI) derived as a conventional surrogate for APD.^6^

Biopsies were taken from an alternans-susceptible and an alternans-resistant site in 17 patients for RNA profiling, and from a further group of seven patients for western blot analysis. In another group of seven patients, after conducting incremental pacing for inducing alternans electrical restitution properties at alternans-susceptible and alternans-resistant sites were studied using a standard S1S2 stimulation protocol.^7^ In this group, no biopsies were collected.

### On-site signal processing to guide myocardial biopsy

Immediately following the pacing protocol, data were exported from the recording system to a PC for on-site analysis. Signal processing was performed by custom-designed algorithms. Activation time (AT) and repolarization time (RT) at each electrode site were measured from the unipolar electrograms as the time of the minimum first derivative during depolarization and the time of the maximum first derivative in both positive and negative T-waves, respectively.^6^ Activation-recovery intervals were calculated as ARI = RT − AT (*Figure 1*). Action potential duration alternans was identified as being present when beat-to-beat variation of ARI exhibited an alternating pattern (long, short, long, short…) for at least eight beats with a magnitude of variation equal or greater than 4 ms.^8^ Colour-coded maps were created which indicated the magnitude of APD alternans over the epicardium. The electrograms from the regions exhibiting highest magnitude of APD alternans and no APD alternans/variability were inspected to ensure high signal quality and were highlighted as targets for biopsy. The entire analysis was carried out within 2 min. Numbered tags on each electrode enabled the precise localization of the site chosen for biopsy with the epicardium. Tru-Cut needle biopsies (CareFusion, San Diego, CA, USA) were taken from the thicker walled left ventricular (LV) sites. To ensure consistency, all biopsies were collected by the same experienced surgeon and tissue technician sectioning the biopsy.

Additionally, for each cardiac site, spatial gradients of repolarization were measured during pacing as the absolute ARI difference between neighbouring sites divided by their distance, averaged across all neighbouring sites within a search radius of 15 mm (see Supplementary material online).

### Electrical restitution analysis

To study the interaction between APD alternans and APD restitution properties,^7^ a standard S_1_–S_2_ pacing protocol was conducted twice per patient, pacing adjacent to an alternans-resistant and an alternans-susceptible site, respectively. The protocol consisted of eight beat trains at basic cycle length S_1_–S_1_ = 600 ms followed by a premature test stimulus S_2_ with decreasing S_1_–S_2_ coupling interval. The S_1_–S_2_ interval was decremented by 50 ms steps from 550 ms to 350 ms and then by 10 ms from 330 ms until loss of ventricular capture. Activation time, RT, and ARI were carefully reviewed and corrected if needed. The diastolic interval (DI) preceding the post extra-stimulus beat was calculated as the difference between local cycle length and RT (see Supplementary material online, *Methods* for details), and restitution curves relating DI and post extra-stimulus ARI were created. Maximal restitution slope, *α*, was calculated using a piecewise linear fitting strategy by performing linear regression in sliding windows 70-ms wide and recording the line with the maximal slope.^9^ Only sites that activated within the first 40 ms were considered in order to minimize possible attenuation of the slope along the pathway of activation due to engagement with conduction velocity restitution.

### RNA isolation

Total RNA was isolated from liquid N_2_ snap frozen biopsy tissue using a mirVana kit (Applied Biosystems) according to the manufacturer’s standard protocol and DNase I treatment. Samples were homogenized for 1 min with an Ika T10 homogenizer (IkaWerke). The concentration of total RNA obtained from each sample was measured using a Nanodrop ND1000 spectrophotometer (Thermo Scientific).

### Reverse transcription

Single-stranded cDNA was synthesized from 144 ng total RNA using High-Capacity RNA-to-cDNA Master Mix (Applied Biosystems) in a 20 μL reaction according to the manufacturer’s protocol. Samples were run on a qPCRVeriti 96 well thermal cycler (Life Technologies).

### qPCR data analysis

qPCR was performed using Taqman low density array (TLDA) microfluidic cards with 48 targets per sample (48a format) (Applied Biosystems) and QuantiTect primer assays (Qiagen) with Power SYBR green fluorescent reporter (Applied Biosystems). Details are given in the Supplementary material online, Methods.

### Histology and western blot

Immunohistochemical staining was performed on frozen sections and western blotting on frozen ventricular biopsy samples. Western Blots were conducted on samples from seven patients for Phospholamban and SERCA2 and from a subset of five patients for CSQN.

### Mathematical modelling

The O’Hara–Rudy model of an undiseased human ventricular cell^10^ was implemented to simulate the electrical action potentials of both alternans-resistant and alternans-susceptible cells based on experimental data. The model was updated to include CSQN-mediated luminal gating of the RyR, which regulates intracellular Ca^2+^ cycling by altering RyR inactivation kinetics (see Supplementary material online, *Methods* for details and references). This modified O’Hara–Rudy model was then taken as control for the alternans-resistant cells. For simulation of alternans-susceptible cells, the model was further modified to incorporate statistically significant changes in RNA observed *in vivo* associated with *I*_K1_, sarcoplasmic Ca^2+^ release (flux) (J_rel_), *I*_to_, the RyR, and CSQN (Supplementary material online, *Table S3*). It was then assumed that a difference in RNA expression between the two tissue regions would result in a corresponding change in the conductance (or maximum activity) of the corresponding ionic channel (or intracellular concentration flux). The single cell model was then incorporated into a two-dimensional ventricular tissue model, taking into consideration the electrotonic coupling between cells. In the tissue model, patches of alternans-susceptible cells were coupled to surrounding patches of alternans-resistant cells. The size of the alternans-susceptible patches were chosen based on the experimental observation, and the patch size was varied to ascertain if a minimum patch size was necessary for production of alternans.

### Statistical analysis

Results of qPCR analysis were divided in alternans-susceptible and alternans-resistant groups. No RNA group of data was normally distributed (Lilliefors test and one-sample Kolmogorov–Smirnov test). Differences in any single RNA between these two groups were therefore quantified with the two-sided Wilcoxon signed-rank test. In order to select the main determinants of APD alternans, only RNA with *P* < 0.01 for at least two out of three housekeepers (18S&HPRT, 18S, and 18S&GAPDH) were considered as significant.

Differences in the magnitude of repolarization gradients between alternans-susceptible and resistant sites were assessed with the two-sided Wilcoxon signed-rank test.

Differences in restitution properties between alternans-susceptible and alternans-resistant sites were quantified by comparing median restitution slope α at alternans-susceptible and alternans-resistant sites with the two-sided paired Wilcox signed-rank test. Differences were assessed by pooling data from the two restitution protocols per patient together and the Holm–Bonferroni correction was used to reduce the probability of Type I errors.

Standard box-plots were used to describe data distribution, where central line is the median, the edges of the box are the first (Q_1_) and third (Q_3_) quartiles and the Whiskers extend to the most extreme data points not considered outliers. Values lower than Q_1_ − 1.5 × (Q_3_ − Q_1_) and higher than Q_3_ + 1.5 × (Q_3_ − Q_1_) are considered outliers.

## Results

### Spatio-temporal organization of alternans and interaction with gradients and restitution properties

**Figure 2:**
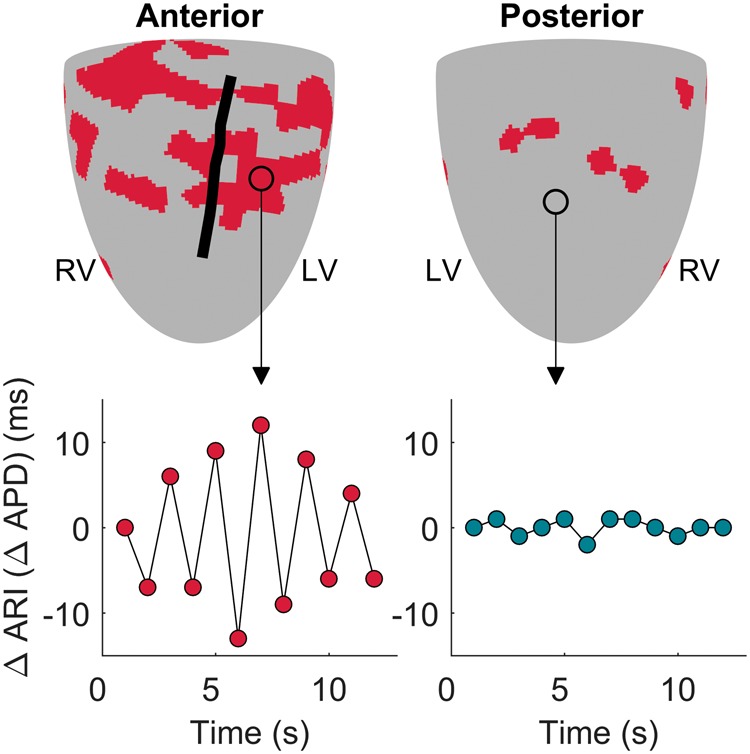
Mapping APD alternans. Map of alternans-susceptible sites (red) in one representative patient’s heart (upper panels). The black line represents the left anterior descending artery. Lower panels show beat-to-beat ARI fluctuations at an alternans-susceptible (left) and at an alternans-resistant (right) site. APD, action potential duration; ARI, activation-recovery interval; LV, left ventricle; RV, right ventricle.

**Figure 3:**
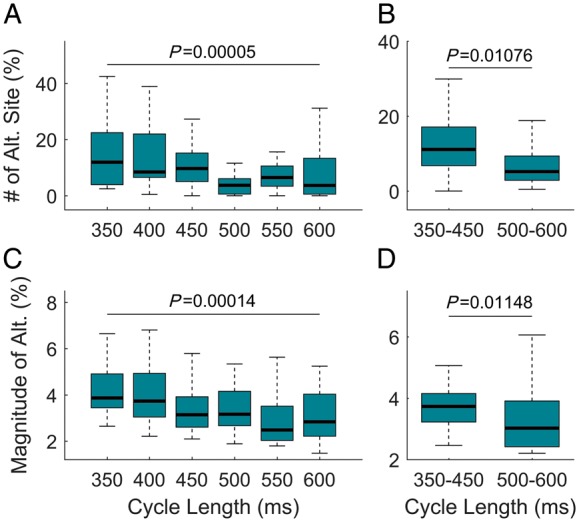
Spatio-temporal distribution of APD alternans. Alternans and cycle length. (*A*) Number of sites showing alternans relative to the total number of sites at a given CL. (*B*) Mean number of alternans-susceptible sites for CL ≤450 ms and CL ≥500 ms. (*C*) Magnitude of alternans relative to the mean ARI at a given CL. (*D*) Mean magnitude of alternans relative to mean ARI for CL ≤450 ms and CL ≥500 ms. *P*-values for group (Kruskal–Wallis test) and pairwise (Wilcoxon signed-rank test) are reported (*n* = 30). APD, action potential duration; ARI, activation-recovery interval; CL, cycle length.

Representative examples of activation and repolarization maps as well as of epicardial recordings exhibiting repolarization alternans are shown in Supplementary material online, *Figures S1–S5*. APD alternans was spatially heterogeneous in all patients (e.g. *Figure 2*). There was a trend for the number of sites exhibiting alternans as well as for the magnitude of alternans relative to the mean ARI to increase at shorter cycle lengths (*P* < 0.05, Kruskal–Wallis for group differences, *Figure 3A and C*). Furthermore, the mean number of alternans-susceptible sites and the average magnitude of alternans relative to the mean ARI was higher for cycle length ≤450 ms than for cycle length ≥500 ms (*P* < 0.05, Wilcoxon signed-rank test, *Figure 3B *and* D*). The prevalence of APD alternans presented a border-line significant trend to increase with shorter APD (Supplementary material online, *Figure S6*), as differences in the number of alternans susceptible-sites within ARI quintiles was significant for cycle lengths ≥500 ms (*P* = 0.047, Kruskal–Wallis test) and elevated but non-significant for cycle lengths ≤450 ms (*P* = 0.078, Kruskal–Wallis test). No significant differences were found in the prevalence of APD alternans within quintiles of AT (Supplementary material online, *Figure S7*) demonstrating that sites activating early showed similar probability of developing APD alternans than sites activating late. No left ventricle/right ventricle, base/apex, or anterior/posterior differences were found in the number of alternans-susceptible sites or in the alternans and gradients magnitudes.

No differences were observed in the prevalence of repolarization alternans between patients with and without reduced LV ejection fraction or between patients with and without previous myocardial infarction (Supplementary material online, *Figure S8*).

**Figure 4:**
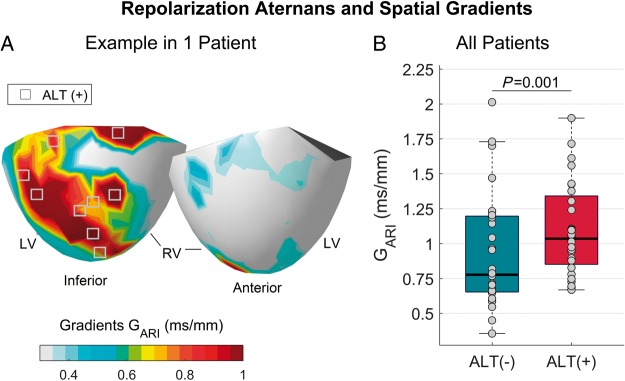
Interaction between alternans and spatial gradients of repolarization. (*A*) The magnitude of spatial gradients of repolarization is colour-coded in a representative patient’s heart. Grey squares represent alternans-susceptible sites and are predominantly located in red regions, indicating large repolarization gradients. (*B*) Mean spatial gradients of repolarization (G_ARI_) in alternans-susceptible, ALT (+), as compare to alternans-resistant sites, ALT (−). Each point shows the mean intra-patient repolarization gradient measured by averaging the gradients across all alternans-susceptible and resistant sites. The box shows the 25th–75th percentile range and the horizontal red line the inter-subject median. Differences were statistically significant (*P* = 0.001, Wilcoxon signed-rank test). APD, action potential duration; ARI, activation-recovery interval; LV, left ventricle; RV, right ventricle.

The magnitude of spatial gradients of repolarization at alternans-susceptible and resistant sites was compared (*Figure 4A*). The mean spatial gradients were significantly larger in alternans-susceptible (1.03, 0.85–1.34 ms/mm, median Q1–Q3) than in alternans-resistant sites (0.77, 0.65–1.2 ms/mm, *P* = 0.001, *Figure 4B*), confirming a potentially pro-arrhythmic interaction between APD alternans and spatial heterogeneity of repolarization.

**Figure 5:**
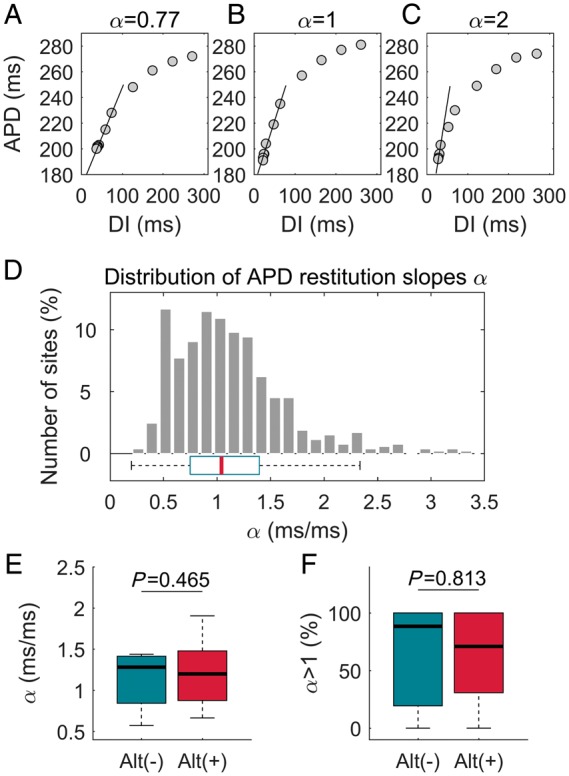
APD alternans and cardiac restitution properties. (*A*–*C*) Representative examples of ARI restitution curves illustrating wide range of APD restitution slopes, *α*, within the same heart. (*D*) Distribution of *α* pooling together all sites which activate within the first 40 ms from all restitution protocols. (*E* and *F*) Relationship between APD alternans and APD restitution slope: there was no difference either in *α* (*E*) or in the proportion of sites with *α *> 1 (*F*) between alternans-susceptible and alternans-resistant sites. Box-plots represent the median *α* at alternans-susceptible and alternans-resistant sites that activate within the first 40 ms (*n* = 7, data from two restitution protocols per patient pooled together). APD, action potential duration; ARI, activation-recovery interval; DI: diastolic interval.

*Figure 5A*–*C* shows a representative example of ARI restitution curves from a same heart illustrating spatial heterogeneity. When pooling together results from all patients and restitution protocols, the median APD restitution slope, *α*, in sites that activated within the first 40 ms was equal to 1.05 (*Figure 5D*), indicating that APD restitution slope higher than one was prevalent. There was no difference either in the steepness of the restitution curves (*Figure 5E*) or in the number of curves with maximum slope *α* >1 between alternans-resistant and alternans-susceptible sites (*Figure 5F*) with *α* equal to 1.28 (0.84–1.41) vs. 1.20 (0.88–1.48), *P* = 0.47, [median (Q1–Q3)], respectively, and the proportion of sites showing α >1 equal to 88% (19–100%) vs. 71% (31–100%), *P* = 0.81, respectively.

### Expression of ion channels and related molecules in the alternans-susceptible and alternans-resistant regions

Total RNA was isolated from pairs of alternans-susceptible and alternans-resistant biopsies from the left ventricle of 17 patients. Biopsies from four patients were excluded from the analysis because of insufficient signal quality in at least one of the two sites.

Biopsies were equally distributed between apical and basal regions, indicating that the results reported below stem from an alternans susceptible/resistant difference and not intrinsic base/apex differences.

**Figure 6:**
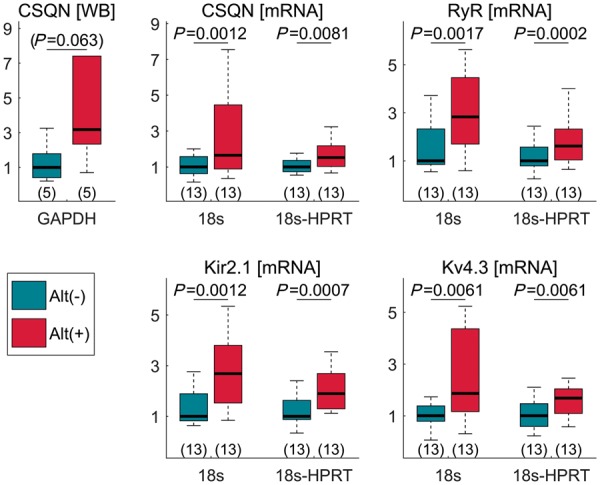
RNA and protein expression analysis. RNA data expression and WB analysis at the alternans-susceptible (red bar) and alternans-resistant (blue bar) sites. Data are normalized to mean value at alternans-resistant sites, which is equal to 1. Housekeepers are indicated below each graph. The sample size for each group is reported in brackets under each boxplot. WB, western blot; CSQN, calsequestrin; RyR, ryanodine.

The most significant changes were observed in the Ca^2+^ handling proteins RyR and CSQN as well as in K_ir_2.1 and K_v_4.3, whose expression was higher in alternans-susceptible than in alternans-resistant samples (*Figure 6*). These differences were highly significant and consistently maintained over different housekeepers (Supplementary material online, *Table S2*). The median relative increase in the alternans-susceptible sample with respect to the alternans-resistant one was always higher than 50%. RyR is the Ca^2+^ release channel of the sarcoplasmic reticulum (SR), whereas CSQN is an SR Ca^2+^ binding protein. K_v_4.3 is a voltage-gated channel involved in the regulation of the transient outward K^+^ current, and Kir2.1 is the principal inward-rectifier K^+^ channel responsible for the inward-rectifier K^+^ current.

Western blot analysis was performed for CSQN, SERCA2, and phospholamban. All samples from alternans-susceptible sites had higher CSQN expression than samples from alternans-resistant sites (Supplementary material online, *Figure S9*), but the number of samples analysed (*N* = 5) was insufficient to reach statistical significance (*P* = 0.06, *Figure 6*). No significant differences were found in phospholamban (*P* = 0.38) and SERCA2 (*P* = 0.22) protein expression.

### Extracellular matrix and histological analysis

Expression of RNAs for some extracellular matrix components (collagen type 1 α1, fibronectin, vimentin, and TGF-β1) was measured, but no significant differences were observed (Supplementary material online, *Table S2*). This suggests that the amount of extracellular matrix and the degree of any fibrosis in the alternans-susceptible and alternans-resistant regions was similar. This was confirmed by histology: tissue sections through alternans-susceptible and alternans-resistant biopsies were stained with Masson’s trichrome and picrosirius red and there was no difference in the amount of extracellular matrix in the two (Supplementary material online, *Figure S10*).

### Biophysical modelling of cellular and tissue behaviour

**Figure 7:**
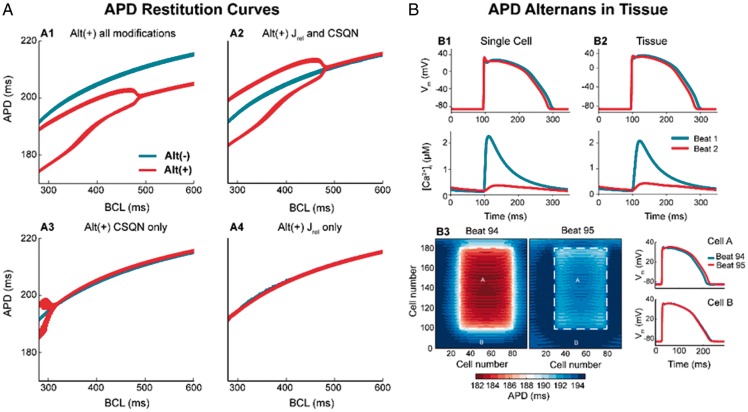
Simulation study. (*A*) APD restitution curves showing the reliance on substrate modification for alternans promotion. Full remodelling (A1) as well as J_rel_ and CSQN modification (A2) result in APD alternans across the same cycle lengths (bifurcation of the red line), while modifications of only CSQN (A3) or J_rel_ (A4) does not promote APD alternans. (*B*) Comparison of AP and Ca^2+^ transient from single cell simulation (B1) and tissue simulation (B2) showing that alternans is preserved in tissue. B3: results of the two-dimensional tissue simulation: a patch of alternans-susceptible cells (patch A, delimited by a white dashed line) surrounded by a larger patch of alternans-resistant cells (patch B) is show for two consecutive beats (beat number 94 and 95). Red and blue represent short and long APD, respectively. A change in APD between the two consecutive beats is observed within the patch of alternans-susceptible cells (Patch A, with red turning light blue) but not in the patch of alternans-resistant cells (Patch B, with blue remaining blue). On the right, action potential traces for both beats from patch A (top) and B (bottom) show that alternans only appears within the patch of alternans-susceptible cells (ΔAPD = 11 ms). APD, action potential duration; CSQN, Calsequestrin; J_rel_, sarcoplasmic Ca^2+^ release (flux).

In single cell simulations, implementation of all four of the observed changes in RNA expression associated with alternans-susceptible tissue resulted in alternans across a range of cycle lengths, between 280 ms and 480 ms. *Figure*7.A1 shows the APD restitution curve from the alternans-resistant cell (blue) showing the characteristic pattern of APD shortening at short cycle length. The restitution curve from the alternans-susceptible site (red) shows a bifurcation for cycle length lower than 480 ms, indicating APD alternans. The functional impact of the changes observed *in vivo* was dissected by modifying model parameters for each substrate individually and in specific combinations. Analysis demonstrated that the greater expression of CSQN and RyR (i.e. J_rel_) may account for the production of alternans, as modification of these two substrates alone produces alternans of the same magnitude as the full modification condition (*Figure*7.*A2*). Greater CSQN expression is determined to be the critical factor in producing alternans, with an increase in J_rel_ enhancing the susceptibility to alternans in combination with CSQN increase, but insufficient to produce alternans on its own (*Figure*7.*A3* and *A4*). Ionic current modifications accounted for the difference in APD observed between the full modification and modified CSQN-RyR only conditions (*Figure*7.*A2*), but did not significantly affect alternans.

Observed APD alternans were driven by secondary effects on ionic current kinetics resulting from intracellular Ca^2+^ alternans (*Figure*7.*B1*). As a result, electrotonic interactions did not significantly affect the vulnerability to alternans, and in tissue simulations, significant alternans was observed throughout the patch of alternans-susceptible tissue, uninhibited by the absence of alternans in the surrounding alternans-resistant tissue (*Figure*7.*B2* and *B3*). Alternans was not significantly influenced by the size of the alternans-susceptible tissue patch.

**Figure 8:**
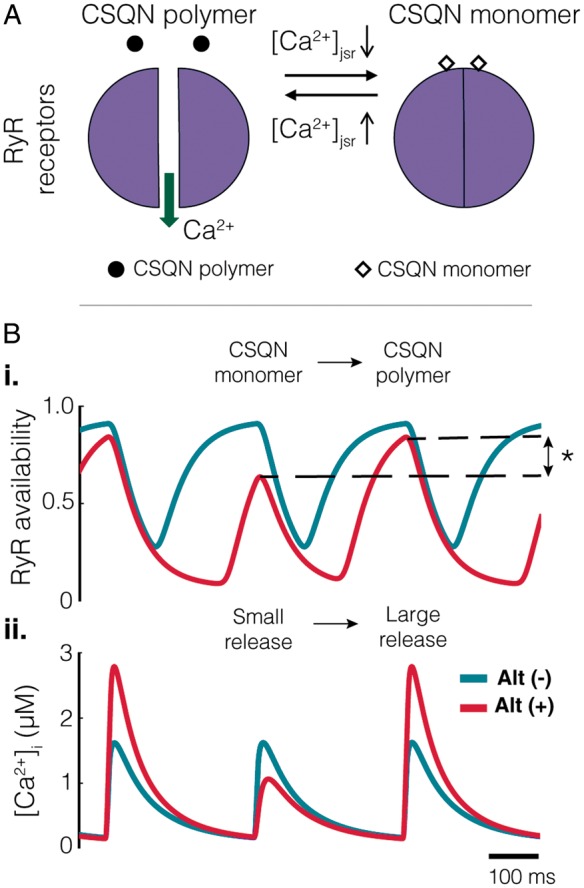
Mechanisms of alternans generation. (*A*) At high [Ca^2+^]_jsr_ CSQN polymerizes and unbinds from the RyRs. This results in a lifting of its inhibition on the RyRs and allows their opening. As [Ca^2+^]_jsr_ decreases, CSQN monomers form and bind to the RyR inhibiting their opening. (*B.i*) A 30% difference in total RyR availability between excitations (denoted by asterisk) results in the small Ca^2+^ release observed. (*B.ii*) The cycle of Ca^2+^ release from the jSR can be observed in the difference in the magnitude of the Ca^2+^ transient between excitations. CSQN, Calsequestrin; RyR, ryanodine.

Greater expression of both CSQN and RyR plays a synergistic role in alternans production (*Figure 8*). Greater abundance of SR Ca^2+^ release mediated by the RyR causes greater SR Ca^2+^ depletion during excitation, resulting in a larger fraction of CSQN monomers, and a prolonged RyR recovery period. Greater expression of CSQN results in a greater number of CSQN monomers at low [Ca^2+^]_jsr_. Thus during Ca^2+^ influx the time taken for polymerization of CSQN is longer with the concomitant increase in the refractory period of the RyR. At shorter cycle lengths this incomplete recovery from inactivation of the RyR leads to incomplete Ca^2+^ release from the jSR. Both of these effects combined to promote established mechanisms of intracellular Ca^2+^ alternans^11^ (*Figure 8*).

## Discussion

Integrating human intact heart mapping with targeted ion channel profiling provided insights into mechanisms of repolarization alternans in the human heart *in vivo*. The main findings were (i) following an incremental pacing protocol electrophysiological mapping revealed a patchy distribution of alternans with areas of alternans interspersed with areas showing no alternans. (ii) The myocardial tissue collected *in vivo* demonstrated significantly greater expression in the calcium handling RNA transcripts CSQN and RyR in alternans-susceptible as compared to alternans-resistant sites, and CSQN protein expression of a limited number of separate samples showed a parallel trend. (iii) The biophysical model utilising the RNA expression data predicted alternans of similar magnitude and in the same range of cycle lengths observed *in vivo*, principally determined by local greater abundance of CSQN and RyR. (iv) Electrical restitution properties (i.e. steepness APD restitution slopes >1) as well as the degree of histological fibrosis between alternans-susceptible and resistant sites were not different, whereas spatial gradients of repolarization were greater in alternans-susceptible than in alternans-resistant sites.

Overall, the distribution of AT and RT (see Supplementary material online, *Figures S1–S4*) was consistent with previous detailed studies on Langendorff perfused human hearts.^12^

Theoretical studies and experimental models have suggested the existence of a causal link between cardiac restitution properties and repolarization alternans.^13^ However, in this study, restitution slopes were not significantly different between alternans-susceptible and resistant regions (*Figure 5*) suggesting that this mechanism is unlikely to be of prime importance. This is in agreement with studies in humans showing no relation between T-wave alternans in ECG and APD restitution slope in LV and RV endocardial sites,^2^ and between APD restitution slope and APD alternans in isolated rabbit hearts.^14^ The latter study highlighted the importance of APD dispersion and conduction velocity restitution.^14^ Similar results were demonstrated *in silico*,^15^ and other studies confirm our finding that APD restitution slope >1 is prevalent in the human heart.^2^ Furthermore, in this study, computational modelling based on mapping guided RNA expression data predicted APD alternans in the same range of cycle lengths as APD alternans observed *in vivo,* which coincided with the plateau rather than the steep portion of the restitution curve.^2^

Previous studies have linked repolarization alternans with calcium handling proteins. For example, a theoretical model has been advanced which relates the properties of local intracellular Ca^2+^ release to whole cell Ca^2+^ alternans referred to as 3R theory.^16^ This unifying framework predicts how Ca^2+^ proteins, RyR, SERCA, NCX L type Ca^2+^ channels and mitochondria, and Ca^2+^ load interact with the parameters in the model and the initiation of APD alternans, one of the key components being RyR refractoriness. A recent study using optical mapping of SR Ca^2+^ in Langendorff rabbit hearts also identified RyR refractoriness as a major mechanism in alternans.^17^ Our combined *in vivo* mapping, RNA expression and *in silico* modelling data predict that APD alternans is partially due to the greater expression of CSQN and RyR and the modulatory role of CSQN on RyR activation kinetics (*Figures 6 and 7*), i.e. refractoriness, and are consistent with these studies.^16^^,^^17^ The computational model therefore suggests a causal link between intracellular Ca^2+^ alternans and repolarization alternans at the tissue level. Furthermore, elegant animal studies have demonstrated that pharmacological restoration of RyR function reduces alternans susceptibility. For instance, Zamiri *et al*.^18^ showed that Dantrolene sodium stabilizes cardiac calcium cycling, increases calcium alternans threshold, and significantly reduces RyR-dependent diastolic calcium leak, therefore, increasing the resistance to malignant arrhythmias.^18^

CSQN is a low-affinity, high-capacity Ca^2+^-binding protein that can store Ca^2+^ within the SR. Each molecule of CSQN can bind 18–50 Ca^2+^ ions. CSQN is also believed to regulate the activity of RyR Ca^2+^ release channels by controlling the local luminal Ca^2+^ concentration in the vicinity of the RyR channels.^19^ Regional differences in Ca^2+^ alternans have also been described in adenoviral gene transfection experiments promoting cellular up-regulation of CSQN in the endoplasmic reticulum and perinuclear regions of cardiomyocytes.^20^ The present study expands our knowledge and understanding of the role of calcium cycling in the genesis of repolarization alternans. In addition to imbalanced SR Ca^2+^ uptake and release, the modulation of RyR kinetics mediated by CSQN can also play a role in generating alternans.

### Limitations

This study presents limitations. The results are based on an heterogeneous population and epicardial mapping and did not include transmural conduction and repolarization dynamics, as simultaneous high-density mapping of epicardial, endocardial, and transmural extracellular potentials is currently not feasible in the context of cardiac surgery. We have primarily focused on transcript data, with borderline supportive protein analysis for CSQN from a limited number of samples, as there are only limited antibodies available to study Ca^2+^ handling proteins and a number of technical challenges. Although biopsies were collected transmurally, whereas electrograms were recorded epicardially, we took care to identify the subepicardial portion of the biopsy at the time of taking it, and only this portion was used for the laboratory analysis. Although the dynamic restitution protocol has been shown to better correlate with APD alternans than the standard S_1_–S_2_ protocol performed in this study,^21^ it also takes considerably longer, and was therefore, incompatible with our clinical set-up and the time available to collect the data in the operation. Since a significant proportion of the study population had ischaemic heart disease, ischaemia may have contributed to the establishment of alternans. The interaction between ischaemia, ionic current remodelling, and APD alternans warrant further investigation. Finally, the amplitude of repolarization alternans induced by electrical stimulation was modest (3–7% of ARI) and in line with previous studies.^8^^,^^21^ It was not possible to directly assess how vulnerable to arrhythmia the alternans-susceptible sites were since the experimental protocol did not include attempting to induce VT/VF and no VT/VF was spontaneously induced. However, repolarization alternans is known to be proarrhythmic and our observation that spatial gradients of repolarization are larger in alternans-susceptible sites as compared to alternans-resistant sites reinforces this notion. Re-entrant arrhythmia may be facilitated by the interaction between spatial heterogeneity in ion channel expression, repolarization alternans, and repolarization gradients that we have documented in presence of elevated heart rate. The elucidation of the exact causal link between these factors warrants further investigation.

## Conclusions

Our data obtained by combining epicardial intact heart electrophysiology, ion channel expression, and computational modelling in human implicate calcium cycling mechanisms, specifically CSQN interactions with RyR, in the mechanism underlying APD alternans. We were unable to find evidence for a role of APD restitution and documented an interaction between repolarization alternans and spatial gradients. These results add human data to experimental models in the search for therapeutic targets to prevent alternans and sudden cardiac death.

## Supplementary Material

euz007_Supplementary_Material

## References

[1] Merchant FM, Armoundas AA. Role of substrate and triggers in the genesis of cardiac alternans, from the myocyte to the whole heart: implications for therapy. Circulation 2012;125:539–49. 22271847 10.1161/CIRCULATIONAHA.111.033563 PMC3281422

[2] Narayan SM, Franz MR, Lalani G, Kim J, Sastry A. T-wave alternans, restitution of human action potential duration, and outcome. J Am Coll Cardiol 2007;50:2385–92. 18154963 10.1016/j.jacc.2007.10.011

[3] Verrier RL, Klingenheben T, Malik M, El-Sherif N, Exner DV, Hohnloser SH et al Microvolt T-wave alternans: physiological basis, methods of measurement, and clinical utilityconsensus guideline by international society for Holter and noninvasive electrocardiology. J Am Coll Cardiol 2011;58:1309–24. 21920259 10.1016/j.jacc.2011.06.029 PMC4111570

[4] Edwards AG, Louch WE. Species-dependent mechanisms of cardiac arrhythmia: a cellular focus. Clin Med Insights Cardiol 2017;11. doi:10.1177/1179546816686061. 10.1177/1179546816686061 PMC5392019 28469490

[5] Taggart P, Orini M, Hanson B, Hayward M, Clayton R, Dobrzynski H et al Developing a novel comprehensive framework for the investigation of cellular and whole heart electrophysiology in the *in situ* human heart: historical perspectives, current progress and future prospects. Prog Biophys Mol Biol 2014;115:252–60. 24972083 10.1016/j.pbiomolbio.2014.06.004

[6] Coronel R, de Bakker JMT, Wilms-Schopman FJG, Opthof T, Linnenbank AC, Belterman CN et al Monophasic action potentials and activation recovery intervals as measures of ventricular action potential duration: experimental evidence to resolve some controversies. Heart Rhythm 2006;3:1043–50. 16945799 10.1016/j.hrthm.2006.05.027

[7] Franz MR. The electrical restitution curve revisted: steep or flat slope—which is better? J Cardiovasc Electrophysiol 2003;14:S140–7. 14760916 10.1046/j.1540.8167.90303.x

[8] Pruvot EJ, Katra RP, Rosenbaum DS, Laurita KR. Role of calcium cycling versus restitution in the mechanism of repolarization alternans. Circ Res 2004;94:1083–90. 15016735 10.1161/01.RES.0000125629.72053.95

[9] Orini M, Taggart P, Srinivasan N, Hayward M, Lambiase PD. Interactions between activation and repolarization restitution properties in the intact human heart: *in vivo* whole-heart data and mathematical description. PLoS One 2016;11:e0161765. 27588688 10.1371/journal.pone.0161765 PMC5010207

[10] O'Hara T, Virag L, Varro A, Rudy Y. Simulation of the undiseased human cardiac ventricular action potential: model formulation and experimental validation. PLoS Comput Biol 2011;7:e1002061. 21637795 10.1371/journal.pcbi.1002061 PMC3102752

[11] Weiss JN, Nivala M, Garfinkel A, Qu Z. Alternans and arrhythmias: from cell to heart. Circ Res 2011;108:98–112. 21212392 10.1161/CIRCRESAHA.110.223586 PMC3076605

[12] Opthof T, Remme CA, Jorge E, Noriega F, Wiegerinck RF, Tasiam A et al Cardiac activation–repolarization patterns and ion channel expression mapping in intact isolated normal human hearts. Heart Rhythm 2017;14:265–72. 27737802 10.1016/j.hrthm.2016.10.010

[13] Qu Z, Xie Y, Garfinkel A, Weiss JN. T-wave alternans and arrhythmogenesis in cardiac diseases. Front Physiol 2010;1:154. 21286254 10.3389/fphys.2010.00154 PMC3028203

[14] Banville I, Gray RA. Effect of action potential duration and conduction velocity restitution and their spatial dispersion on alternans and the stability of arrhythmias. J Cardiovasc Electrophysiol 2002;13:1141–9. 12475106 10.1046/j.1540-8167.2002.01141.x

[15] Cherry EM, Fenton FH. Suppression of alternans and conduction blocks despite steep APD restitution: electrotonic, memory, and conduction velocity restitution effects. Am J Physiol Heart Circ Physiol 2004;286:H2332–41. 14751863 10.1152/ajpheart.00747.2003

[16] Qu Z, Nivala M, Weiss JN. Calcium alternans in cardiac myocytes: order from disorder. J Mol Cell Cardiol 2013;58:100–9. 23104004 10.1016/j.yjmcc.2012.10.007 PMC3570622

[17] Wang L, Myles RC, De Jesus NM, Ohlendorf AK, Bers DM, Ripplinger CM. Optical mapping of sarcoplasmic reticulum Ca^2+^ in the intact heart: ryanodine receptor refractoriness during alternans and fibrillation. Circ Res 2014;114:1410–21. 24568740 10.1161/CIRCRESAHA.114.302505 PMC4000583

[18] Zamiri N, Massé S, Ramadeen A, Kusha M, Hu X, Azam MA et al Dantrolene improves survival after ventricular fibrillation by mitigating impaired calcium handling in animal models. Circulation 2014;129:875–85. 24403563 10.1161/CIRCULATIONAHA.113.005443

[19] Knollmann BC. New roles of calsequestrin and triadin in cardiac muscle. J Physiol (Lond) 2009;587:3081–7. 19451205 10.1113/jphysiol.2009.172098 PMC2727016

[20] Guo A, Cala SE, Song LS. Calsequestrin accumulation in rough endoplasmic reticulum promotes perinuclear Ca^2+^ release. J Biol Chem 2012;287:16670–80. 22457350 10.1074/jbc.M112.340927 PMC3351355

[21] Koller ML, Maier SKG, Gelzer AR, Bauer WR, Meesmann M, Gilmour JRF. Altered dynamics of action potential restitution and alternans in humans with structural heart disease. Circulation 2005;112:1542–8. 16157783 10.1161/CIRCULATIONAHA.104.502831

