# Supplementary material for: Mechanistic insights from targeted molecular profiling of repolarization alternans in the intact human heart
Source: Europace. 2019 Feb 8;21(6):981–9. doi: 10.1093/europace/euz007 (PMC6545501; doi:10.1093/europace/euz007)
Supplement: euz007_Supplementary_Material [file euz007_supplementary_material.pdf]

SUPPLEMENTAL MATERIAL

Mechanistic insights from targeted molecular profiling of repolarization alternans in the intact human heart

Michele Orini[a,b*], PhD, Joseph Yanni[c*], PhD, Peter Taggart[a*], MD, PhD, Ben Hanson[d], PhD, Martin Hayward[e], MD, Andrew Smith[b], MD, Henggui Zhang[c,f], PhD, Michael Colman[g], PhD, Gareth Jones[f], PhD, Xiao Jie[a], PhD, Halina Dobrzynski[c], PhD, Mark R Boyett[c], PhD, Pier D Lambiase[a,b], MD, PhD

<u>*****Joint first authors</u>

Contents

1. Supplemental Methods 3

   1.1. Analysis of electrophysiological data 3

   1.2. Electrical restitution analysis 4

   1.3. Histological analysis 5

   1.4. qPCR data analysis 6

   1.5. Western blotting 7

   1.6. Mathematical Modelling 7

   References 10

2. Supplemental Tables 11

   2.1 Supplemental Table 1 11

   2.2. Supplemental Table 2 13

   2.3. Supplemental Table 3 14

   2.4. Supplemental Table 4 15

   2.5. Supplemental Table 5 17

   2.6. Supplemental Table 6 17

   2.7. Supplemental Table 7 17

3. Supplemental Figures 18

Supplemental Figure 1 18
Supplemental Figure 2 19
Supplemental Figure 3 20
Supplemental Figure 4 21
Supplemental Figure 5 22
Supplemental Figure 6 23
Supplemental Figure 7 24
Supplemental Figure 8 25
Supplemental Figure 9 26
Supplemental Figure 10 27
Supplemental Figure 11 28
Supplemental Figure 12 29

1. Supplemental Methods

1.1. Analysis of electrophysiological data

Signal processing was performed by custom-designed algorithms which included: (a) Removal of pacing artefact with minimal distortion; (b) Filtering for noise reduction (third order Butterworth filter with a band pass between 0.5 Hz and 25 Hz); (c) Quantitative assessment of the quality of the recording from each electrode both in terms of signal-to-noise ratio (SNR) and morphological consistency between consecutive waveforms.

SNR was estimated from the pre-filtered signals from each electrode and after removing the pacing artefacts as:

$$SNR = 10\ \log_{10}\frac{P_s}{P_n}\ [dB]$$

where P_s and P_n represent power content in the signal spectral band [0.1-40 Hz] and in the noise spectral band [40 – 100 Hz]. Traces with a SNR higher than 15dB were considered of sufficient quality for analysis.

Morphological consistency was quantified by computing the average of the Pearson correlation coefficients, ρ, between a template representing the average filtered waveform during a heartbeat at a given cycle length, $\overline{V(t)}$, and each single waveform, $V_i(t)$. The waveform was defined as $V_i(t) = V(t)$ with $t=[t_i, t_i+T]$, where $V(t)$ is the filtered unipolar electrogram, t_i is the time of delivering the ith S_1 stimulus and T is the fixed cycle length. Thus, ρ was calculated as:

$$\rho = \frac{1}{N}\sum_{i=1}^{N}\frac{Cov(V_i(t),\overline{V(t)})}{\sigma_{V_i(t)}\sigma_{\overline{V(t)}}},\ \text{and}\quad \overline{V(t)} = \frac{1}{N}\sum_{i=1}^{N}V_i(t)$$

where $i=\{1,\ldots,N\}$ are heartbeats with same cycle length.

Within each heartbeat, activation (AT) and repolarization (RT) times were estimated from the inflection points in the unipolar electrograms. AT corresponds to the time from the trigger event (pacing stimulus) to the minimum of the first derivative, $AT_i = \arg(\min(dV_i(t)/dt))$, during depolarization phase, while RT corresponds to the time from the pacing stimulus to the maximum of the first derivative, $RT_i = \arg(\max(dV_i(t)/dt))$, during the repolarization phase for both positive and negative T-waves. Activation recovery interval (ARI) was then calculated as $ARI_i = RT_i - AT_i$. APD alternans was identified as being present whenever the beat-to-beat variation of ARI, $\Delta ARI = ARI_i -$

$ARI_{i-1,}$ exhibited an alternating pattern (long, short, long, short …) for at least 8 beats with a magnitude of variation ($mean(|\Delta ARI_i|)$) equal or higher than 4ms. APD alternans was discarded if activation time also exhibited an alternating pattern. If more than one period of alternans was identified for a given electrode site and within the same drive train, the highest-magnitude period was chosen to represent the electrode site.

Alternans was quantified for every paced cycle length drive train and the maximal magnitude across all cycle lengths was used as representative of a given epicardial site.

Spatial gradients of repolarization were measured at each cardiac site, j, and for each beat, i, as:

$$G_j^{(i)} = \frac{1}{K}\sum_{k=1}^{K} \frac{|ARI_j - ARI_k|}{d_{jk}}$$

This expression represent the as the absolute ARI difference between neighbouring sites, $k=\{1,\ldots,K\}$, divided by their distance, d_{jk}, averaged across all neighbouring sites, K, within a given search radius D, i.e. $d_{jk}<D$.

For each patient, the mean intra-patient gradients within alternans-susceptible, $G_{ALT(+)}$, and alternans-resistant, $G_{ALT(-)}$, sites were computed as:

$$G_{ALT(+)} = \frac{1}{N \times J_{ALT(+)}}\sum_{i}^{N}\sum_{j=1}^{J_{ALT(+)}} G_j^{(i)}$$

$$G_{ALT(-)} = \frac{1}{N \times J_{ALT(-)}}\sum_{i}^{N}\sum_{j=1}^{J_{ALT(-)}} G_j^{(i)}$$

where $J_{ALT(+)}$ and $J_{ALT(-)}$ are the total number of alternans-susceptible and resistant sites and N the total number of paced beats included in the calculation, which are all of them independent of the cycle length but excluding the first 5 beats after increasing cycle length. Comparison between mean intra-patient gradients within alternans-susceptible and resistant sites was performed using Wilcoxon signed-rank test.

1.2. Electrical restitution analysis

A standard S_1-S_2 pacing protocol was conducted twice per patient, pacing adjacent to an alternans-resistant and an alternans-susceptible site, respectively. Following each train of nine steady-state S1 stimuli, a shorter interval S2 stimulus was interposed. The S_1–S_2 interval was decremented by 50 ms steps from 550 to 350 ms; then by 20 ms to 330 ms; and then by 10 ms intervals until loss of ventricular capture. AT, RT and ARI were calculated as described in the previous section, carefully reviewed and corrected if needed. Diastolic interval (DI) preceding the S2 beat was obtained as DI =

$CL_{S1\text{-}S2}$ - ARI_{med}, where $CL_{S1\text{-}S2}$ is the local cycle length preceding the S2 beat and measured as the interval between the local activation time of the last drive beat and the local activation time of beat S2; ARI_{med} is the median ARI at basic cycle length, which was computed for each drive train as the median ARI of the last 5 beats at basic cycle length.

Maximal restitution slope, α, was calculated using a piecewise linear fitting strategy by performing linear regression (least square algorithm) in sliding windows 70-ms wide and recording the line with the maximal slope. Exponential models, $ARI = ARI_{max} - ae^{-(1/b*DI)}$, were also fitted to the data (least square Levenberg-Marquardt algorithm) to describe DI-ARI dynamics. Only restitution curves with a coefficient of determination $R^2 > 0.6$, and meeting the previously described inclusion criteria of signal-to-noise ratio and morphological stability were included. Only epicardial sites which activated within the first 40 ms (i.e. those close to the pacing site) were considered in order to minimise the attenuating effect of slowed activation time limiting the minimum DI achievable at distal sites. Differences in restitution properties between alternans-susceptible and alternans-resistant sites were quantified by comparing median α at alternans-susceptible and resistant sites with the two-sided paired Wilcox signed-rank test. Differences were assessed by pooling data from the two restitution protocols per patient together. In case of P<0.05, the Holm-Bonferroni correction was used to reduce the probability of type I errors (incorrect rejection of a true null hypothesis).

1.3. Histological analysis

Masson's trichrome staining was performed on frozen sections. Sections were fixed in Bouin's fluid (Sigma Aldrich) overnight. Next, sections were washed in 70% ethanol three times (10 min each wash), stained in celestine blue for 5 min, and rinsed in distilled water. Sections were then stained in Mayer's alum hematoxylin for 10 min, washed in tap water for 15 min, stained in acid fuchsin for 3 min, and rinsed in distilled water. Next, sections were treated with phosphomolybdic acid for 5 min, drained, stained in methyl blue for 5 min, rinsed in distilled water, and treated with 1% acetic acid for 2 min, followed by dehydration through alcohols (1 min 70% ethanol, 1 min 90% ethanol, 2×2 min 100% ethanol). Finally, sections were placed in Histoclear (2×5 min) and mounted in DPX mounting medium (Fluka). Images of sections were taken using light microscopy (Carl Zeiss Microscopy) using Axiovision software (Carl Zeiss Microscopy). With this technique, connective tissue was stained royal blue, cardiac myocytes were stained pink, and nuclei were stained dark blue. For picrosirius red staining, sections were fixed in 10% neutral buffered formalin for 30 min at room temperature. Sections were then washed in water three times (10 min each wash). Sections were placed into picrosirius red solution for 1 h at room temperature. After that, sections were washed in acetic acid water twice (10 min each wash). Then sections were dehydrated by placing them in

different concentrations of alcohol as follows: 1 min in 70 % alcohol, 1 min in 90 % alcohol and 2 min in absolute alcohol. After dehydration, the sections were placed in clear solvent (xylene) twice (5 min each). Lastly, the sections were mounted in DPX mounting medium. Images of tissue sections were collected using a polarising microscope (collagen fibres appear red). High magnification images (×20 for picrosirius red staining) were collected using the same microscope parameters. 10-15 images per region per patient were collected. Velocity software (Improvision, UK) was used to measure the signal intensity (in arbitrary units) for each protein. Signal intensity was only measured in user defined regions.

1.4. qPCR data analysis

qPCR was performed using two methods: (i) Taqman low density array (TLDA) microfluidic cards with 48 targets per sample (48a format) (Applied Biosystems) used with an ABI Prism 7900HT Sequence Detection System (Applied Biosystems). A list of the assays on the TDLA cards is shown in Supplemental Table 4. 144 ng total RNA converted to cDNA in 50 µl water was loaded into each sample reservoir with 50 µl Taqman Universal Master Mix (Applied Biosystems). (ii) QuantiTect primer assays (Qiagen) with Power SYBR green fluorescent reporter (Applied Biosystems). This method was used to look at a number of extra targets (Supplemental Table 5) in 10 µl reactions using the ABI Prism 7900HT Sequence Detection System.TLDA cards were analysed using RQ manager (Applied Biosystems) and RealTimeStatMiner (Integromics). Average threshold cycle (Ct) values were obtained using RQ manager, and amplification curves were analysed to check for experimental errors. Reactions that failed quality analysis were removed from further analysis. Expression levels were calculated using the ΔCt method with 18S&HPRT, 18S, 18S&GAPDH used as a housekeeper. RealTime StatMiner software was used to analyse the data from the TLDA cards and the GeNorm stability score method was used to examine the suitability of the potential housekeepers for use individually and also in combination. Biological replicate outliers were removed using StatMiner via the use of MAD unit comparison, with samples with >3 flags being removed from the analysis. Data obtained using QuantiTect primer assays were analysed using SDS 2.4 (Applied Biosystems) to obtain Ct values and the efficiency (E) of PCRs. 28S was used as the housekeeper for ΔCt expression level calculation. Outliers were determined using a robust statistical modified z-score method based on the median of absolute deviation and were excluded from the statistical analysis. Data were calculated as $E^{-\Delta Ct}$.

1.5. Western blotting

Ventricular samples from both alternans-susceptible and alternans-resistant tissue samples were dissected and flash frozen in liquid N_2 and stored at -80°C. Protein was isolated by homogenising tissue in RIPA buffer (10 ml PBS, 10 ml 10% IGEPAL CA-630 [Sigma Aldrich], 5 ml 10% Na deoxycholate [Sigma Aldrich], 1 ml 10% SDS [BDH], 0.5 ml 100 mmol/L PMSF [Sigma Aldrich], 50 µl 1 mg/mL leupeptin [Sigma Aldrich], 50 µl 2 mg/mL aprotinin [Sigma Aldrich], 0.25 µl 1 mg/ml pepstatin [Sigma Aldrich], and 73.15 ml deionised water). Homogenate was then centrifuged at 3000 rpm for 5 min at 4°C, and the supernatant was collected. Protein yield was quantified using Qubit protein assay (Life Technologies). 36 µg of protein was used for each sample, and the volume was increased to 7.5 µl with deionised water. To each sample, 2.5 µl NuPAGE LDS sample buffer was added. Samples were loaded onto NuPAGE 4% to 12% bis-tris gels (Life Technologies). NuPAGE MOPS SDS running buffer (Life Technologies) was used, and electrophoresis was run at 200 V constant for 50 min. Western blot onto Immobilon-P PVDF membrane (Millipore) was performed using a transfer buffer consisting of 100 ml 10× running buffer (30 g Tris base [144 g glycine in 1 l deionised water], 200 ml methanol, 700 ml deionised water) and run at 30 V constant for 2 h. Transfer was checked by staining membranes with Ponceau red solution for 5 min, followed by rinsing in deionised water. After staining, membranes were cleared by washing for 30 s in 0.1 M NaOH solution. Membranes were blocked in 3% BSA (Sigma Aldrich) in TBS-T (100 ml TBS 10× [24.23 g TrizmaHCl, 80.06 g NaCl in 1 l deionised water, pH 7.6], 900 ml deionised water, 1 ml Tween20 [Sigma Aldrich]) overnight at 4°C. Membranes were washed 3 times in TBS-T followed by staining with primary antibody diluted in 1% TBS-T to the appropriate concentration (Supplemental Table 5) for 2 h at room temperature. Membranes were washed 3 times in TBS-T. Appropriate HRP-conjugated secondary antibody was diluted to the required concentration (Supplemental Table 6) in 1% BSA in TBS-T applied to membranes for 2 h at room temperature. Membranes were washed 3 times in TBS-T. Amersham ECL detection reagents (GE Healthcare) were used to visualise the signal using ChemiDoc MP imaging system (Bio-Rad). Band density was quantified using Image Lab 3.0.1 (Bio-Rad) and normalized to the expression of GAPDH and α-tubulin to correct for variation in protein loading. Paired t-test was performed to find statistical differences in expression. $P<0.05$ was taken to be significant.

1.6. Mathematical Modelling

The O'Hara-Rudy (ORd) model of an undiseased human ventricular cell (1) was implemented to simulate the electrical action potentials of both alternans-resistant and alternans-susceptible cells based on experimental data. The model was updated to include CSQN mediated luminal gating of the

RyR (2), which regulates intracellular Ca^{2+} cycling by altering RyR inactivation kinetics.

Such a CSQN-RyR regulation involves the following processes: At low junctional sarcoplasmic reticulum (jSR) Ca^{2+} concentrations ($[Ca^{2+}]_{jsr}$), CSQN may take a monomeric form which binds to the RyR, inhibiting their opening, whereas at higher $[Ca^{2+}]_{jsr}$, CSQN forms polymers that dissociate from the RyR, relieving the inhibition (2). The concentration and dissociation time of CSQN monomers and polymers, respectively, modulate the refractory period of the RyR affecting the intracellular Ca^{2+} release. To simulate such CSQN-RyR regulatory interaction, the O'Hara-Rudy model was modified by the inclusion of a new gate controlling RyR mediated sarcoplasmic Ca^{2+} release, J_{rel}, with kinetics being dependent on the fraction of total sarcoplasmic reticulum (SR) CSQN in monomeric form and the Ca^{2+} dependence of CSQN monomeric/polymeric form consistent with a previous study (3).

More specifically, within the original ORd model CSQN acts as a Ca^{2+} buffer in the jSR. This jSR buffering term is given as:

$$\beta_{jSR} = \frac{1}{1 + \frac{CSQN_{tot} \cdot K_{m,CSQN}}{K_{m,CSQN} + [Ca^{2+}]_{jSR}}}$$

$$CSQN_{tot} = 10\ mM$$

$$K_{m,CSQN} = 0.8\ mM$$

where $CSQN_{tot}$ is the total CSQN concentration and $K_{m,CSQN}$ is the half maximum concentration. From this we can calculate the amount of free CSQN (CSQN not bound to Ca^{2+}) and Ca^{2+} bound CSQN using the equations below.

$$CSQN_{free} = \frac{CSQN_{tot} \cdot K_{m,CSQN}}{K_{m,CSQN} + [Ca^{2+}]_{jSR}}$$

$$CSQN_{bound} = CSQN_{tot} - CSQN_{free}$$

Using the amount of free CSQN we then created a new gating variable that described the amount of monomeric CSQN present in the jSR. This gate was named $monomer_{CSQN}$ and is given by the following equations:

$$monomer_{CSQN,\infty} = \frac{1}{1 + \exp\left(-6.5 \cdot \left(CSQN_{free} - 6.37\right)\right)}$$

$$\tau_{monomer,CSQN} = 20\ ms$$

This transition was the utilised in a second gating variable to simulate the change in open probability of the RyRs with the transition of CSQN from monomeric to polymeric form.

$$RyR_{CSQN} = \frac{1}{1 + \exp\left(5 \cdot \left(monomer_{CSQN} - 0.5\right)\right)}$$

$$\tau_{CSQN} = 50\ ms$$

Due to the lack of quantitative data regarding the time course of monomer to polymer transition and CSQN dissociation from the RyR, time constants were set within empirical physiologically ranges. With the addition of these gates the model was able to simulate the regulation of RyR open probability by CSQN.

The equation regulating RyR release in the ORd model (Equation X) was then updated to include the new gate regulated as follows:

$$Jrel = \left(1.0 - \phi_{rel,CaMK}\right) \cdot Jrel_{np} + \phi_{rel,CaMK} \cdot Jrel_{CaMK}$$

$$Jrel = RyR_{CSQN}\left(\left(1.0 - \phi_{rel,CaMK}\right) \cdot Jrel_{np} + \phi_{rel,CaMK} \cdot Jrel_{CaMK}\right)$$

where $\phi_{rel,CaMK}$ is the fraction of phosphorylated RyR channels, $Jrel_{np}$ is the flux through the non-phosphorylated RyRs and $Jrel_{CaMK}$ the flux through the phosphorylated RyRs and RyR_{CSQN} is the open probability of the CSQN regulated gate.

Simulation protocols: The model was paced for 100 beats at BCLs of 300, 500 and 1000 ms and the AP and CaT compared with that of the unmodified ORd model. No difference was observed in AP or CaT between the two models (Supplemental Figure 11).

For single cell simulations, to reach a steady state the model was paced for 100 beats until stabilisation of the CaT. To simulate APD restitution the model was paced for 100 beats at each BCL using an S1 stimulation protocol and the APD recorded for the last 2 beats. No differences were observed in the APD restitution between the updated (with the inclusion of RyR_{CSQN}) and the unmodified ORd models (Supplemental Figure 12).

Computational implementation: This modified ORd model was then taken as control for the alternans resistant cells. For simulation of alternans susceptible cells, the model was further modified to incorporate statistically significant changes in mRNA expression of proteins responsible for I_{K1}, I_{rel}, I_{to}, the RyR and CSQN. For details, please see Supplemental Table 3. In simulations, we assumed that a difference in mRNA expression between the two tissue regions would result in a corresponding change in the conductance (or maximum activity) of the corresponding ionic channel (or intracellular concentration flux), as implemented in our previous studies (4). The single cell model was then incorporated into a two-dimensional ventricular tissue model, taking into consideration the electrotonic coupling between cells as in previous studies (5). In the tissue model, patches of alternans susceptible cells were coupled to surrounding patches of alternans resistant cells. The size of the

alternans susceptible patches were chosen based on the experimental observation and the patch size was varied to ascertain if a minimum patch size was necessary for production of alternans.

References

1. O'Hara T, Virag L, Varro A, Rudy Y. Simulation of the undiseased human cardiac ventricular action potential: model formulation and experimental validation. PLoS Comput Biol 2011;7:e1002061.
2. Beard NA, Laver DR, Dulhunty AF. Calsequestrin and the calcium release channel of skeletal and cardiac muscle. Prog Biophys Mol Biol 2004;85:33-69.
3. Restrepo JG, Weiss JN, Karma A. Calsequestrin-mediated mechanism for cellular calcium transient alternans. Biophysical Journal 2008;95:3767-3789.
4. Chandler NJ, Greener ID, Tellez JO et al. Molecular architecture of the human sinus node: insights into the function of the cardiac pacemaker. Circulation 2009;119:1562-1575.
5. Adeniran I, El Harchi A, Hancox JC, Zhang H. Proarrhythmia in KCNJ2-linked short QT syndrome: Insights from modelling. Cardiovasc Res 2012;94:66-76.

2. Supplemental Tables

2.1 Supplemental Table 1

Patients information. From left to right: Patient number; Type of study: “ALT+mRNA”= Biopsies were collected from an alternans-susceptible and an alternans-resistant site for mRNA analysis. “ALT+WB”= Biopsies were collected from an alternans-susceptible and an alternans-resistant site for Western Blot analysis. “ALT+Restitution”: Restitution protocols were performed to study the interaction between APD alternans and the slope of the APD restitution curve; Age; Gender; Operation: AVR=Aortic valve replacement; CABGxN = Coronary artery bypass grafting, with N vessels grafted; LVEF<45%: Left ventricular ejection fraction lower than 45%; MI: Presence of a previous myocardial infarction; Diabetes; HT= Hypertension; Hypertrophic = Hypertrophic ventricles.

Pt #	Study	AGE	Gender	Operation	LVEF<45%	MI	Diabetes	HT	Hypertrophic
1	ALT+mRNA	69	M	CABGx2	x	x	x		
2	ALT+mRNA	49	M	CABGx3		x	x		
3	ALT+mRNA	79	M	CABGx3		x	x	x	
4	ALT+mRNA	49	M	CABGx2					
5	ALT+mRNA	74	M	CABGx3				x	
6	ALT+mRNA	62	M	AVR			x	x	x
7	ALT+mRNA	60	M	CABGx3			x	x	
8	ALT+mRNA	31	M	AVR			x	x	
9	ALT+mRNA	75	F	AVR			x	x	x
10	ALT+mRNA	49	M	CABGx2	x		x	x	x
11	ALT+mRNA	80	F	CABGx3		x	x	x	
12	ALT+mRNA	71	M	AVR + CABGx1			x	x	x
13	ALT+mRNA	51	M	CABGx3		x	x	x	
14	ALT+mRNA	47	M	CABGx4			x	x	
15	ALT+mRNA	78	M	CABGx4			x		
16	ALT+mRNA	77	M	CABGx3				x	
17	ALT+mRNA	73	M	CABGx3					
18	ALT+WB	75	M	AVR + CABGx3	x		x	x	
19	ALT+WB	73	M	AVR + CABGx1		x	x	x	x
20	ALT+WB	44	M	CABGx3	x	x	x	x	
21	ALT+WB	54	M	CABGx2			x	x	
22	ALT+WB	84	M	AVR + CABGx3	x		x	x	x

23	ALT+WB	73	M	CABGx3		x	x	x	x
24	ALT+WB	67	M	CABGx2	x	x	x	x	
25	ALT+Restitution	33	M	CABGx2			x	x	
26	ALT+Restitution	81	M	CABGx3			x	x	
27	ALT+Restitution	59	M	CABGx3			x	x	
28	ALT+Restitution	47	M	CABGx3		x	x	x	
29	ALT+Restitution	58	F	AVR			x	x	
30	ALT+Restitution	39	M	AVR	x		x	x	x
31	ALT+Restitution	76	M	CABGx2			x	x	x

2.2. Supplemental Table 2

Transcripts in the alternans-susceptible and alternans-resistant tissue samples. For each transcript, the median relative increase/decrease in alternans-susceptible with respect to alternans-resistant tissue, and the p-value are given (n=13). Differences with P<0.01 (in bold) for at least 2 housekeepers were considered significant.

**Housekeeper**	**18s**		**18s-HPRT**		**18s-GAPDH**	
**mRNA**	**(+)/(-)**	**p-value**	**(+)/(-)**	**p-value**	**(+)/(-)**	**p-value**
**Kir2.1**	2.68	**0.001**	1.89	**0.001**	1.51	**0.006**
**RYR2**	2.83	**0.002**	1.61	**0.000**	1.91	**0.006**
**Calseq2**	1.65	**0.001**	1.52	**0.008**	1.54	0.040
**Kv4.3**	1.86	**0.006**	1.68	**0.006**	1.92	0.013
cholinergic receptor, muscarinic 2	2.09	**0.006**	1.04	0.010	0.90	0.094
Kir6.2	1.36	0.013	1.83	**0.005**	1.45	0.021
Ca(v)β2subunit	1.72	0.010	1.45	0.033	1.43	0.110
Kir6.1	2.43	0.040	1.59	**0.008**	1.85	0.191
NAV1.5	1.60	0.021	1.72	0.027	1.53	0.040
Ca(v)α2δ1	1.49	0.021	1.98	0.033	1.80	0.094
NCX1	2.36	0.021	1.45	0.057	1.58	0.110
SERCA2	1.84	0.021	1.26	0.057	1.34	0.068
PhosphoLamban	2.29	0.033	1.25	0.048	1.10	0.127
adrenoceptor β1	2.39	0.057	1.11	0.080	1.10	0.168
SK1	3.33	0.048	2.08	0.094	6.55	0.040
MinK	1.10	0.010	1.17	0.146	0.74	0.376
Ca(v)Ltypeα1Csubunit	2.58	0.027	1.53	0.146	2.34	0.146
Connexin43	1.72	0.027	1.14	0.146	1.36	0.216
Ca(v)β1subunit	2.44	0.057	1.59	0.127	1.36	0.146
Kir3.4	2.11	0.027	1.40	0.168	1.12	0.146
ATPase, Na+/K+ transporting, α1	1.40	0.057	1.33	0.191	1.18	0.305
inositol 1,4,5-trisphosphate receptor,type 3	2.34	0.146	1.94	0.191	1.85	0.305
NHE1	1.48	0.094	1.83	0.244	1.95	0.273
KCHIP2	3.22	0.068	1.68	0.273	1.10	0.305
PMCA4	1.62	0.110	0.98	0.305	1.04	0.216

KVLQT1	1.30	0.168	1.09	0.340	1.40	0.244
Kir2.3	1.49	0.146	1.71	0.455	1.93	0.305
Cx45	0.97	0.168	1.35	0.455	1.02	0.787
SUR2	1.55	0.244	1.21	0.414	1.17	0.893
Na(v)Ibetasubunit	2.04	0.080	1.36	0.588	1.23	0.946
inositol 1,4,5-trisphosphate receptor 1	0.97	0.273	1.18	0.455	0.87	0.635
adrenoceptor β2, surface	1.40	0.191	1.16	0.542	1.15	0.735
Ca(v)β3subunit	1.60	0.376	2.01	0.414	1.16	0.191
Kir2.2	1.22	0.588	1.09	0.635	1.37	0.635
tyrosine hydroxylase	4.36	0.685	2.36	0.542	1.88	0.455
chloride channel 3	1.42	0.340	1.26	0.946	0.36	0.542
Kv1.5	1.21	0.946	0.88	0.340	1.30	1.000
HERG1	0.89	0.588	1.14	0.839	1.76	0.893
PMCA1	1.13	0.685	1.12	0.839	0.74	0.946
SK3	2.00	1.000	1.11	0.542	1.07	0.735
Kv1.4	1.46	0.685	0.83	0.893	1.00	0.542
Ca(v)γ4subunit	1.01	0.946	0.64	0.635	3.16	0.787
choline acetyltransferase	3.29	1.000	2.35	0.893	1.21	0.946
Kir3.1	0.98	1.000	0.75	1.000	0.72	0.735

2.3. Supplemental Table 3

Scaling factors used to simulate changes in mRNA expression

Protein / Channel	Scaling factor
Kir2.1 / I_{K1}	1.89
RYR2 / I_{rel}	1.61
Kv4.3 / I_{to}	1.68
CSQN	1.52

2.4. Supplemental Table 4

Assays used on TDLA cards.

Target	Description	Assay Number
ABCC9	SUR2	Hs00245832_m1
ADRB1	β1 adrenoceptor	Hs02330048_s1
ADRB2	β2 adrenoceptor	Hs00240532_s1
ATP1A1	α1 subunit of Na^+-K^+ pump	Hs00167556_m1
ATP2A2	SERCA2	Hs00544877_m1
ATP2B1	PMCA1	Hs01001488_m1
ATP2B4	PMCA4	Hs01067999_m1
CACNA1C	Ca_V1.2	Hs00167681_m1
CACNA2D1	$Ca_V\alpha2\delta1$	Hs00984856_m1
CACNB1	$Ca_V\beta1$	Hs00609497_m1
CACNB2	$Ca_V\beta2$	Hs00167861_m1
CACNB3	$Ca_V\beta3$	Hs00167873_m1
CACNG4	$Ca_V\gamma4$	Hs01061935_m1
CACNG7	$Ca_V\gamma7$	Hs00259061_m1
CASQ2	calsequestrin 2	Hs00154286_m1
CHAT	choline acetyltransferase	Hs00252848_m1
CHRM2	muscarinic (M2) receptor	Hs00265208_s1
CLCN3	Cl^- channel, voltage-sensitive 3	Hs00923164_m1
GJA1	Cx43	Hs00748445_s1
GJC1	Cx45	Hs00271416_s1
ITPR1	type 1 IP_3 receptor	Hs00976063_m1
ITPR3	type 3 IP_3 receptor	Hs01573555_m1
KCNA4	K_V1.4	Hs00357903_s1
KCNA5	K_V1.5	Hs00266898_s1
KCND3	K_V4.3	Hs00542597_m1
KCNE1	minK	Hs00264799_s1
KCNH2	ERG1	Hs00542478_m1
KCNIP2	KChIP2	Hs00183958_m1
KCNJ11	K_{ir}6.2	Hs00265026_s1

KCNJ12;KCNJ18	$K_{ir}2.2$	Hs00253248_s1
KCNJ2	$K_{ir}2.2$	Hs00265315_m1
KCNJ3	$K_{ir}3.1$	Hs01002552_m1
KCNJ4	$K_{ir}2.3$	Hs00544821_m1
KCNJ5	$K_{ir}3.4$	Hs00168476_m1
KCNJ8	$K_{ir}6.1$	Hs00958961_m1
KCNN1	SK1	Hs00158457_m1
KCNN3	SK3	Hs00158463_m
KCNQ1	K_vLQT1	Hs00165003_m1
PLN	phospholamban	Hs00160179_m1
RYR2	type 2 ryanodine receptor	Hs00181461_m1
SCN1B	$Na_v\beta1$	Hs00168897_m1
SCN5A	$Na_v1.5$	Hs00165693_m1
SLC8A1	NCX1	Hs01062258_m1
SLC9A1	NHE1	Hs00300047_m1
TH	tyrosine hydroxylase	Hs01002182_m1

2.5. Supplemental Table 5

QuantiTect assays used.

Target	Description	Assay Number
TGFβ1	transforming growth factor beta 1	QT00000728
TNF	Tumour necrosis factor	QT00029162
Collagen type 1 α1	collagen, type I, alpha 1	QT00037793
Fibronectin 1	Fibronectin 1	QT01000839

2.6. Supplemental Table 6

Primary antibodies used.

Protein	Host	Type	Concentration	Manufacturer
SERCA2	Mouse	Monoclonal (IgG)	1:1000	Fisher Scientific
Phospholamban	Rabbit	Polyclonal (IgG)	1:100	Abcam
Calsequestrin	Rabbit	Polyclonal (IgG)	1:1000	Abcam
GAPDH	Rabbit	Polyclonal (IgG)	1:1000	Abcam
α-tubulin	Rabbit	Polyclonal (IgG)	1:1000	Abcam

2.7. Supplemental Table 7

Secondary antibodies used.

Host	Type	Conjugate	Concentration	Manufacturer
Goat	Rabbit (IgG)	HRP	1:2000	Cell signalling Technology
Goat	Mouse (IgG)	HRP	1:2000	Cell signalling Technology

3. Supplemental Figures

Supplemental Figure 1

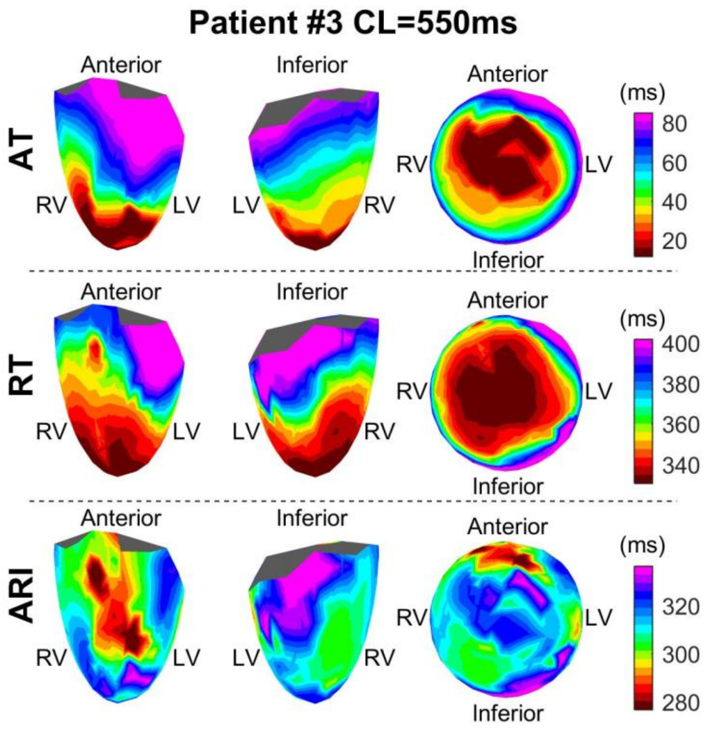

Supplemental Figure 1: Isochrone maps representing activation time (AT, top), repolarization time (RT, middle) and activation recovery interval (ARI, bottom), a standard surrogate for local APD. Each column shows a different view of the same map. Patient 3, CL = 550 ms.

Supplemental Figure 2

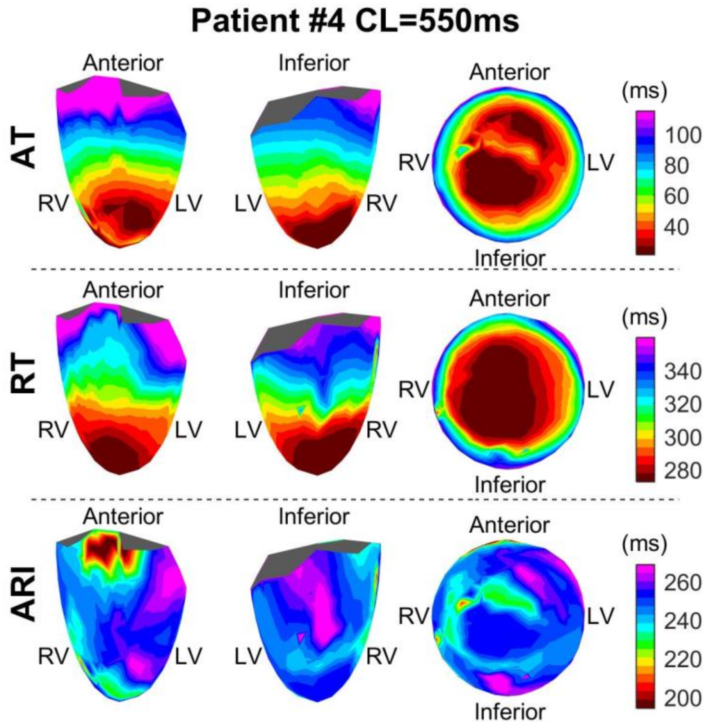

Supplemental Figure 2: Isochrone maps representing activation time (AT, top), repolarization time (RT, middle) and activation recovery interval (ARI, bottom), a standard surrogate for local APD. Each column shows a different view of the same map. Patient 4, CL = 550 ms.

Supplemental Figure 3

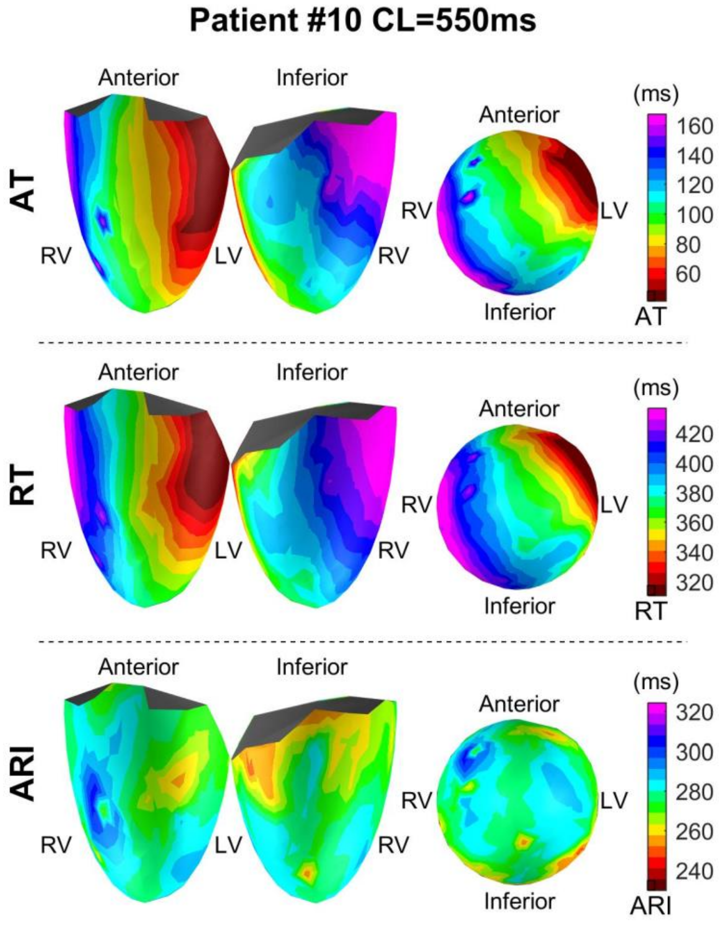

Supplemental Figure 3: Isochrone maps representing activation time (AT, top), repolarization time (RT, middle) and activation recovery interval (ARI, bottom), a standard surrogate for local APD. Each column shows a different view of the same map. Patient 10, CL = 550 ms.

Supplemental Figure 4

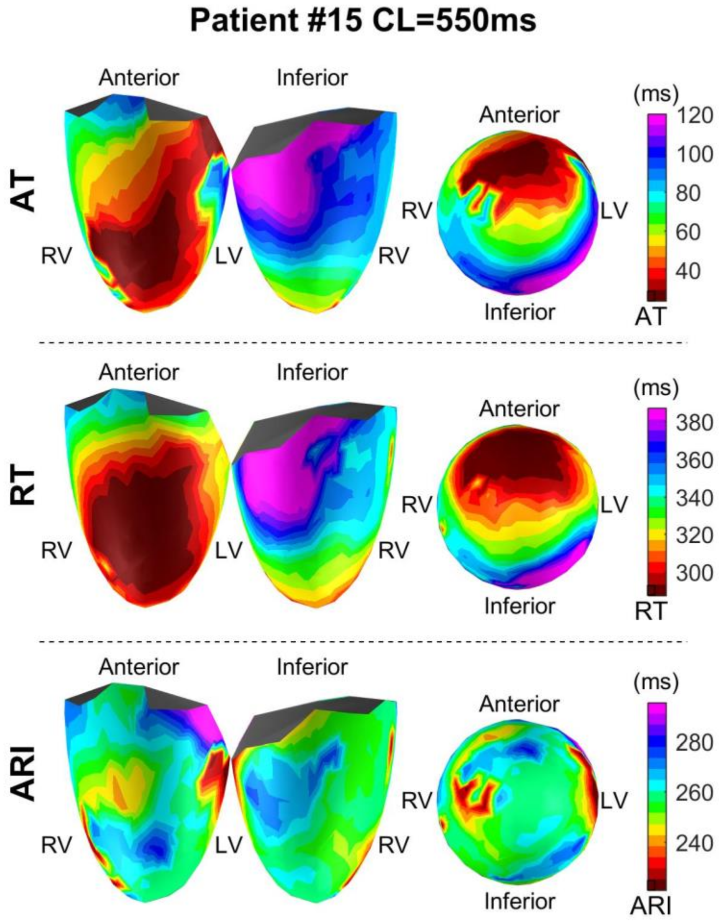

Supplemental Figure 4: Isochrone maps representing activation time (AT, top), repolarization time (RT, middle) and activation recovery interval (ARI, bottom), a standard surrogate for local APD. Each column shows a different view of the same map. Patient 15, CL = 550 ms.

Supplemental Figure 5

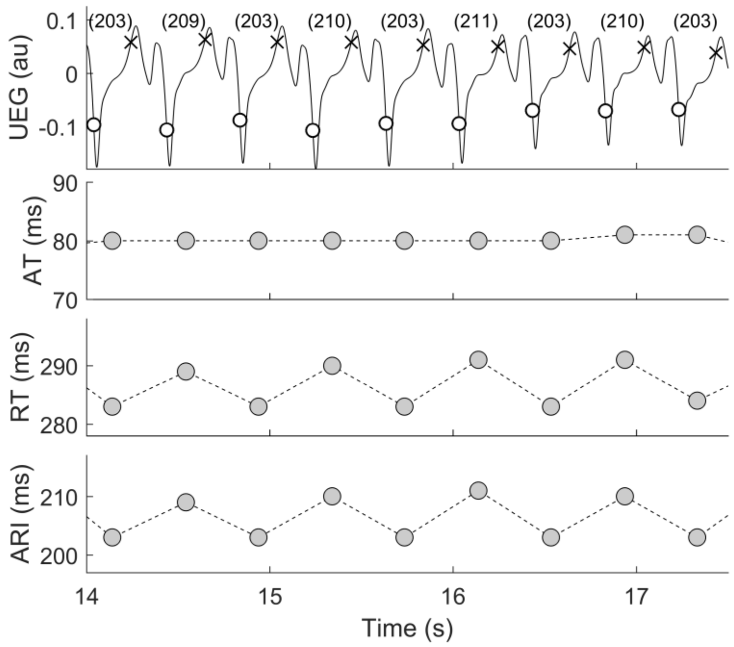

Supplemental Figure 5. APD alternans quantification. A representative unipolar eletrogram (top) with corresponding beat-to-beat activation (AT) and repolarization time (RT), as well as activation-recovery interval (ARI) variability at an alternans susceptible site. ARI is shown in brackets (ms) in the upper panel.

Supplemental Figure 6

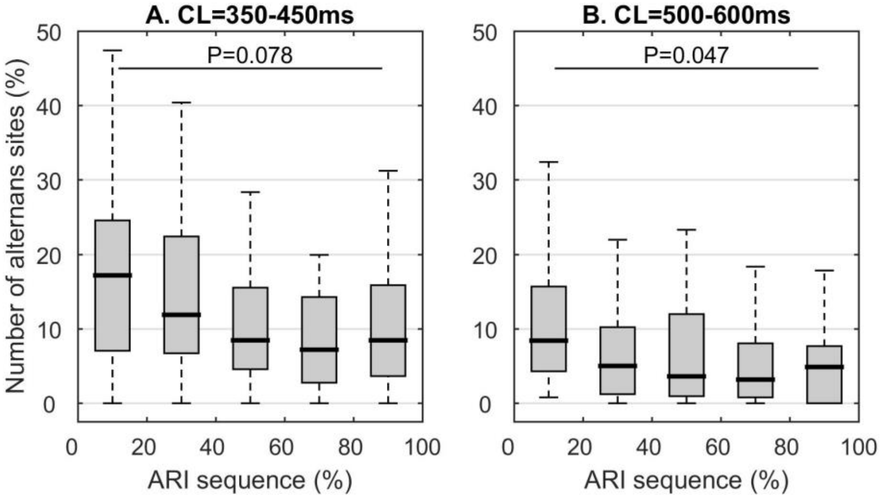

Supplemental Figure 6: Prevalence of APD alternans per ARI quintile. There was a weak trend for APD alternans to occur more often for shorter ARI. The difference in the prevalence of APD alternans within ARI quintiles was not significant for CL≤450 ms (Panel A, P=0.078, Kruskal Wallis test) and weakly significant for CL≥500 ms (panel B, P=0.047, Kruskal Wallis test).

Supplemental Figure 7

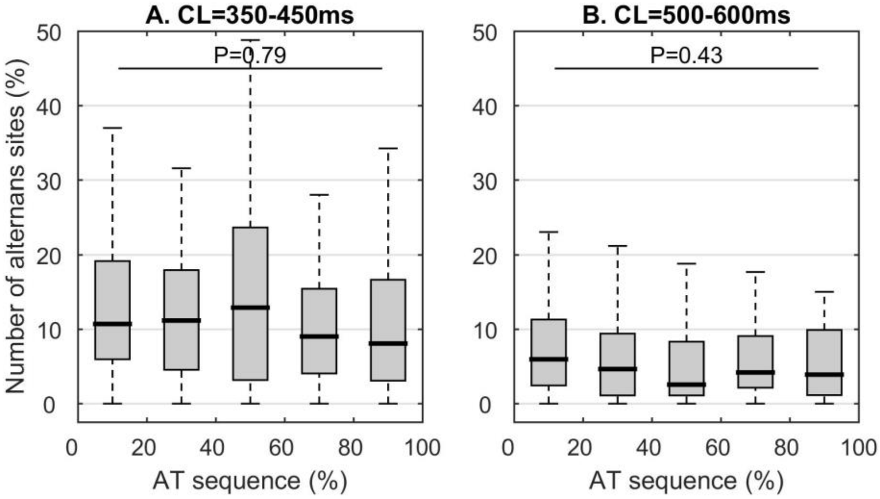

Supplemental Figure 7: Prevalence of APD alternans per ARI quintile. There was no difference in the prevalence of APD alternans for different AT quintiles, demonstrating that sites activating early showed similar probability of developing APD alternans than sites activating late. Panel A and B show results for cycle length equal or lower than 450 ms and equal or higher than 500 ms, respectively. Group statistics performed using Kruskal Wallis test.

Supplemental Figure 8

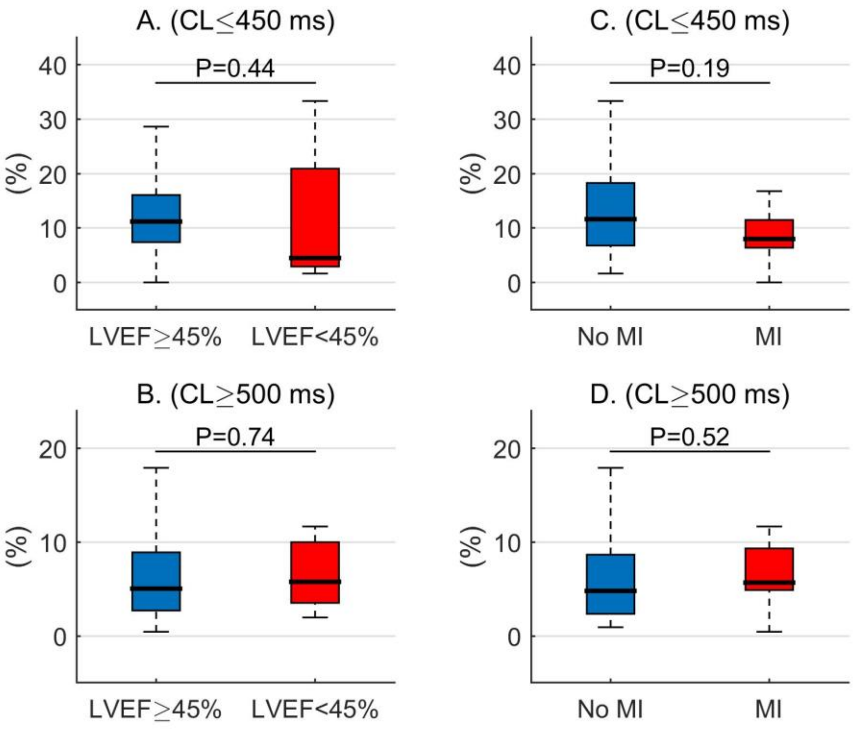

Supplemental Figure 8: Left: Proportion of APD alternans sites in patients with preserved (LVEF≥45%) vs impaired (LVEF<45%) LV ejection fraction for CL≤450 ms (above) and CL≥500 ms (bottom). Right: Proportion of APD alternans sites in patients with vs without previous myocardial infarction for CL≤450 ms (above) and CL≥500 ms (bottom). P-values (Wilcoxon signed-rank test) are reported in each panel. No significant differences were found between the two groups.

Supplemental Figure 9

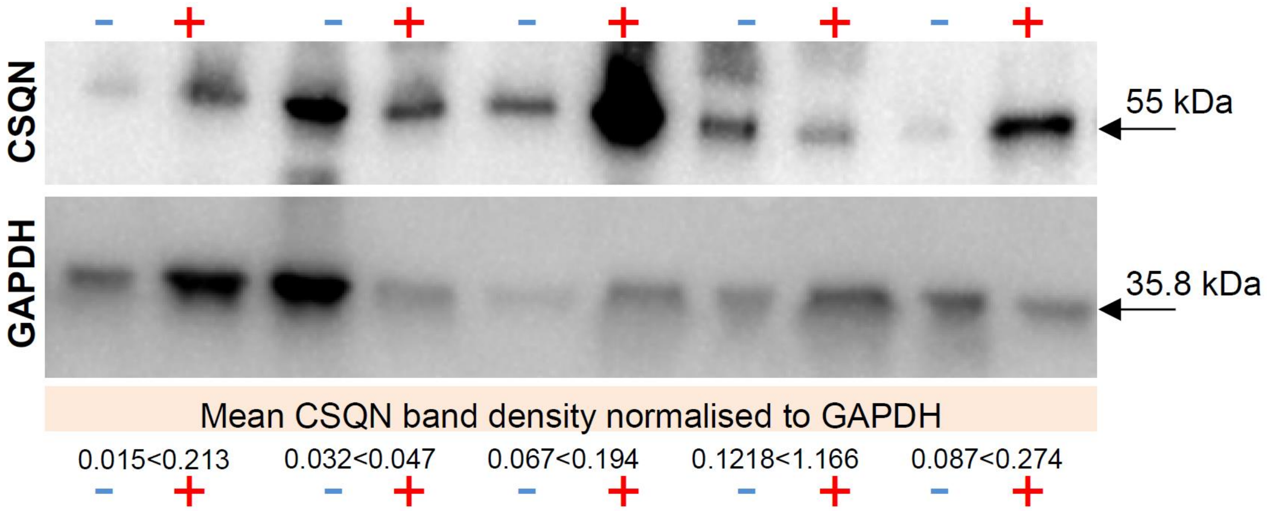

Supplementary Figure 9: Western blot showing protein expression of calsequestrin (CSQN, top) and housekeeper GAPDH (bottom). The mean CSQN band density normalised to GAPDH (from three runs) is reported below the CSQN. This was higher in alternans-susceptible sites (marked as +) than in alternans resistant sites (marked as -) for all patients.

Supplemental Figure 10

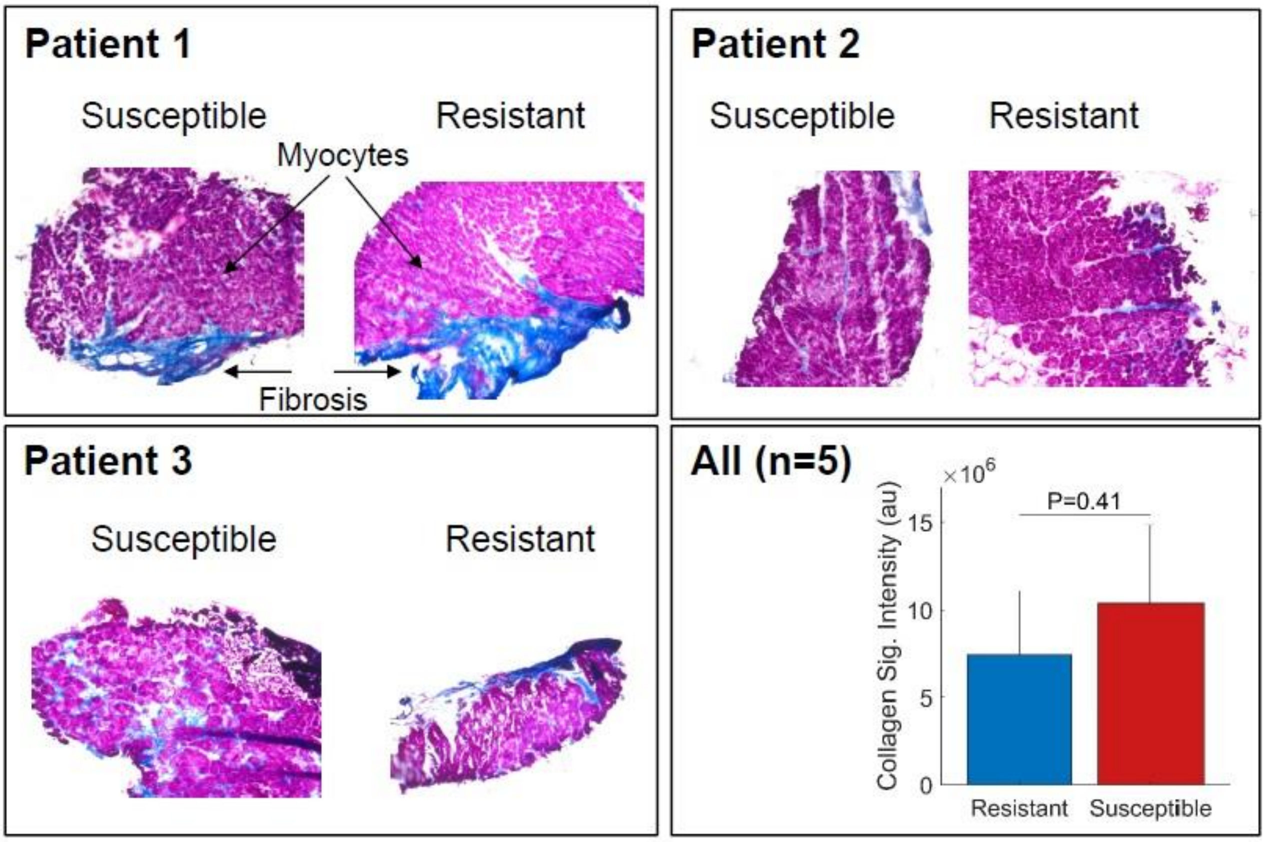

Supplemental Figure 10: Masson's trichome staining of tissue sections from alternans-susceptible (left) and alternans-resistant (right) sites collected from three representative patients. The degree of histological fibrosis between the two sites was equivalent. Myocytes are shown in pink/purple, while connective tissue is shown in blue.

Supplemental Figure 11

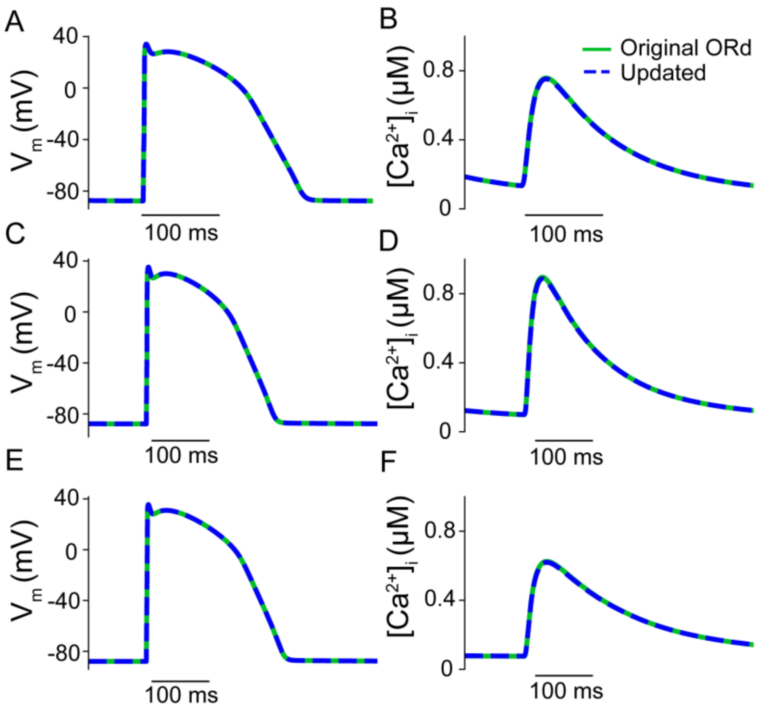

Supplemental Figure 11. A. Comparison between APs produced by the original ORd model (solid green line) and the updated model incorporating CSQN regulation of Jrel (dashed blue line) at a BCL of 300 ms. No difference was observed between the models. B. No difference was observed in the CaT produced by the models at a BCL of 300 ms. C. At a BCL of 500 ms no difference was observed in the AP produced by the models. D. At a BCL of 500 ms the CaT produced by each model was the same. E. At a BCL of 1000 ms no change was observed between the models. F. No difference was observed between the CaT produced by each model.

Supplemental Figure 12

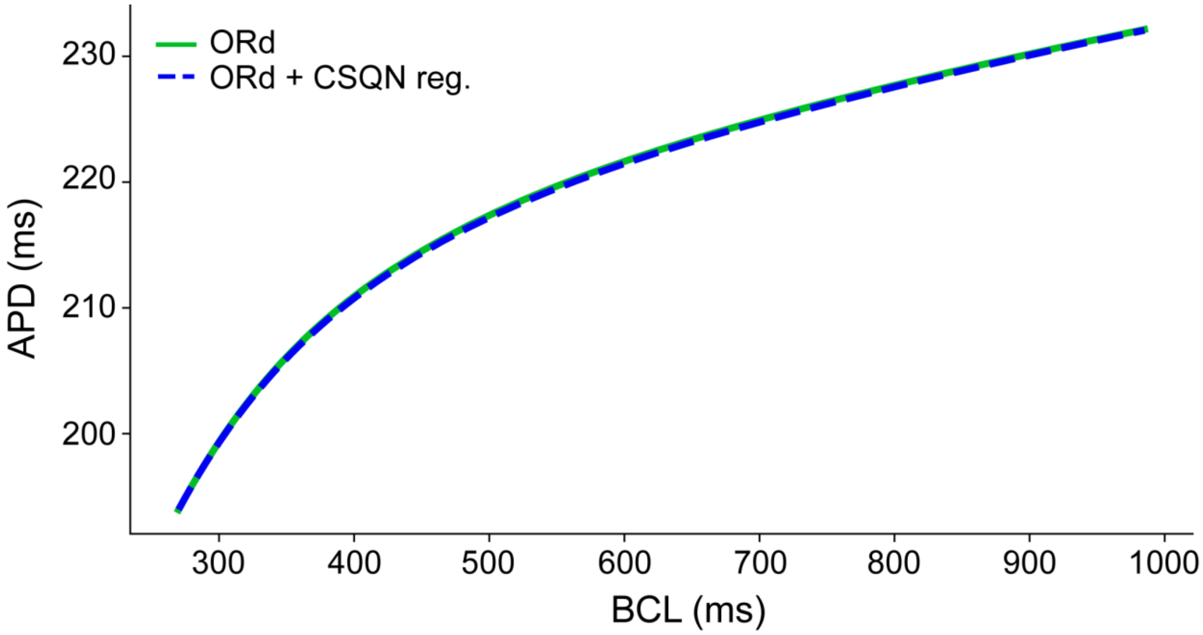

Supplemental Figure 12. AP restitution curve produced by the ORd model (solid green line) and the updated model incorporating CSQN regulation of the RyRs (dashed blue line). No difference was observed between the two models.
